# A New Stochastic Model for Subgenomic Hepatitis C Virus Replication Considers Drug Resistant Mutants

**DOI:** 10.1371/journal.pone.0091502

**Published:** 2014-03-18

**Authors:** Nikita V. Ivanisenko, Elena L. Mishchenko, Ilya R. Akberdin, Pavel S. Demenkov, Vitaly A. Likhoshvai, Konstantin N. Kozlov, Dmitry I. Todorov, Vitaly V. Gursky, Maria G. Samsonova, Alexander M. Samsonov, Diana Clausznitzer, Lars Kaderali, Nikolay A. Kolchanov, Vladimir A. Ivanisenko

**Affiliations:** 1 Department of Systems Biology, Institute of Cytology and Genetics SB RAS, Novosibirsk, Russia; 2 Department of Computational Biology, St. Petersburg State Polytechnical University, St. Petersburg, Russia; 3 Institute for Medical Informatics and Biometry, Technische Universität Dresden, Dresden, Germany; 4 PB-soft Llc, Novosibirsk, Russia; 5 Chebyshev Laboratory, St. Petersburg State University, St. Petersburg, Russia; 6 Theoretical Department, Ioffe Physical-Technical Institute of the Russian Academy of Sciences, St.Petersburg, Russia; University of California, Merced, United States of America

## Abstract

As an RNA virus, hepatitis C virus (HCV) is able to rapidly acquire drug resistance, and for this reason the design of effective anti-HCV drugs is a real challenge. The HCV subgenomic replicon-containing cells are widely used for experimental studies of the HCV genome replication mechanisms, for drug testing in vitro and in studies of HCV drug resistance. The NS3/4A protease is essential for virus replication and, therefore, it is one of the most attractive targets for developing specific antiviral agents against HCV. We have developed a stochastic model of subgenomic HCV replicon replication, in which the emergence and selection of drug resistant mutant viral RNAs in replicon cells is taken into account. Incorporation into the model of key NS3 protease mutations leading to resistance to BILN-2061 (A156T, D168V, R155Q), VX-950 (A156S, A156T, T54A) and SCH 503034 (A156T, A156S, T54A) inhibitors allows us to describe the long term dynamics of the viral RNA suppression for various inhibitor concentrations. We theoretically showed that the observable difference between the viral RNA kinetics for different inhibitor concentrations can be explained by differences in the replication rate and inhibitor sensitivity of the mutant RNAs. The pre-existing mutants of the NS3 protease contribute more significantly to appearance of new resistant mutants during treatment with inhibitors than wild-type replicon. The model can be used to interpret the results of anti-HCV drug testing on replicon systems, as well as to estimate the efficacy of potential drugs and predict optimal schemes of their usage.

## Introduction

Hepatitis C virus (HCV) chronifies in ≈80% of infections, causing hazardous liver diseases, most notably liver cirrhosis and hepatocellular carcinoma. Current success in treating HCV is associated with the use of such drugs as ribavirin, peginterferon, protease inhibitors, and their combinations. However, the drug resistance of virus remains an important problem. Substantial efforts therefore are made to understand the mechanism of HCV infection, as well as to develop new antiviral therapies [1]–[4]. The HCV RNA genome is very heterogeneous because of the high error rate of the viral RNA polymerase NS5B. This is the main reason why the virus rapidly acquires drug resistance [5].The establishment of cell culture systems based on HCV subgenomic replicons is indispensable for analysis of the efficacy of newly developed inhibitors of viral and host proteins that participate in the viral genome replication [6]–[16].

The replication cycle of the HCV subgenomic replicon in Huh-7 cells includes: 1) the IRES-mediated translation of the plus-strand RNA with the formation of polyprotein, processing of which by HCV NS3 protease yields the viral nonstructural proteins NS3, NS4A, NS4B, NS5A, NS5B; 2) the formation of membrane vesicles induced by the viral protein NS4B with the participation of the HCV NS5B protein and the cellular proteins PI4K-IIIα, cyclophilin A/B, hVAP-A/B among others; 3) the replication of the viral genome in membrane vesicles. Inside of the membrane vesicles, the RNA-dependent RNA polymerase NS5B associates with the 3′-end of the viral plus-strand RNA and initiates the *de novo* synthesis of minus-strand RNA. This minus-strand RNA serves as a template for the synthesis of new plus-strand RNA, presumably directly from the double-stranded RNA intermediate. Newly synthesized plus-strand RNA can then either be used for re-initiation of minus strand synthesis, or is exported from the membrane vesicles to the cellular cytoplasm, where it can again be translated or incorporated into membrane vesicles for the next replication round [7], [17]–[19].

The HCV NS3 protease is one of the promising candidates for the design of new potential anti-HCV drugs. New α-ketoamide inhibitors of NS3 protease, namely telaprevir (VX-950) [20], [21], boceprevir (SCH 503034) [22]–[24], narlaprevir (SCH 900518) [25], [26], as well as macrocyclic inhibitors, such as ciluprevir (BILN 2061) [20], [27], [28] and danoprevir (ITMN-191) [29]–[31] have been suggested as promising anti-HCV drugs. These inhibitors impede the processing of viral polyprotein [22], [32] and probably restore the pathways of the innate immune system [32], [33].

It is well known that HCV is able to quickly acquire drug resistance. The drug resistant mutant RNAs appear during the wild-type RNA replication, partly, because the HCV NS5B polymerase lacks a proofreading function [5]. Indeed, mutants that are stable to different inhibitors of NS3 protease were selected *in vitro* [32], [34]–[36] and identified in patients' plasma [32], [37]. The sensitivity of the mutant RNAs to drugs used and their replicative capacity are the two factors that determine mutant selection under the inhibitor action [11], [12], [38], [39].

Two sources of the mutant RNAs that determine the acquisition of drug resistance by virus are described in the literature. The first one is the RNA quasi species that are resistant to a specific drug and preexist both in cell culture and patient liver or plasma prior to therapy [8], [12], [40]–[42]. The other type appears during the wild-type viral RNA replication in the presence of drug [8], [12], [32], [38], [39].

Due to the rapid development of resistance in HCV, increasing attention was recently paid to inhibitors of host factors involved in HCV replication. Cyclophilin A/B, the cellular peptidyl-prolyl*cis/trans* isomerase, that directly interacts with the NS5B and NS5A proteins of the viral replicase complex and stimulates the RNA binding activity of this complex, was recently proposed as a prospective target for antiviral therapeutic strategies [18], [43], [44]. Moreover, it was shown that cyclophilin A plays a critical role in the formation of functional replicase within membrane vesicles [18]. Targeting host factors is advantageous, as they are less prone to select for resistant mutations in the viral genome. Indeed, it was proven to be much more difficult todevelop resistance to cyclophilin inhibitors, namely cyclosporine A or its analogue NIM811, than to NS3/4A protease or NS5B polymerase inhibitors [8], [14], [45], [46].

Mathematical models can improve our understanding of antiviral action and may predict the efficacy of potential drugs. Several models were developed to describe the HCV RNA dynamics and drug effects in patients [47]–[51]. Such models do not consider intracellular processes explicitly or use their simplified presentations only. Despite this fact they exhibit a reasonable accuracy in describing the RNA dynamics in serum [47], [48].

Modeling of the intracellular HCV replication processes appears to be a very complicated mathematical problem. Dahari H. et al. [52] developed the first mathematical model of the subgenomic hepatitis C virus replication in Huh-7 cells. The model includes known stages of the replicon replication, namely translation, protein maturation, and replication of the plus and minus RNA strands in the generalized vesicle. The model considers the number of active ribosomes restricting the otherwise unlimited viral RNA production as a cellular factor. The model allows to correctly describe viral RNA replication in the absence of inhibitors and establishment of the steady state after transfection. We proposed a mathematical model of the suppression of the subgenomic hepatitis C virus RNA replication [53]. This model was aimed to evaluate the effect of drugs on viral RNA replication. An integral cellular factor stands for both known [18], [19] and unknown host proteins involved in the formation of active replicase responsible for the synthesis of the viral plus- and minus-strand RNAs; it restricts the number of active replicases and, as a consequence, the number of viral RNA copies at the steady state.

Binder et al. [54] recently suggested extended model of Dahari [52] in particular by inclusion of generalized cell factor like in Mischenko et al. [53].The model made it possible to explain the differences in HCV permissiveness in different cell lines.

Nakabayashi J. [55] proposed a compartmentalized mathematical model of the replication process of the full size HCV genome, including the assembly of new viral particles in a single infected cell. The model was formulated to study the effect of compartmentalization on viral RNA replication and virion accumulation. MacLean and co-authors [56], also focused on the HCV full life cycle, including translation, replication and assembly. In contrast to the model from Mishchenko et al. [53], none of these models included the action of drugs on viral or cellular targets and none of them were used to describe viral RNA kinetics in the presence of inhibitors. However, the model from Mishchenko et al. [53] failed to simulate the dynamics of inhibitor action for time intervals longer than 3 days.

Here we develop a minimal stochastic mathematical model, describing the kinetics of the viral RNA suppression by the NS3 protease inhibitors in replicon cells. In contrast to the previous model [53], the new one provides a good description of the long-term dynamics of viral RNA inhibition in the presence of inhibitors. It turned out that the resistant mutants need to be taken into account for the correct description of the long-term kinetics. We considered the well known inhibitors of NS3 protease, namely BILN-2061, VX-950, SCH-503034, SCH-900518, and ITMN-2061 that are expected to be promising for anti-HCV drugs design [20], [22], [25], [29]. Experimental data for the kinetics of viral RNA inhibition are available for three of them (BILN-2061, VX-950, and SCH-503034) [20], [22]. HCV mutants resistant to these inhibitors exist, and their replication capacity, as well as sensitivity to inhibitors are known [11], [38]. We showed that in order to get a good agreement between the model and the kinetics of viral RNA inhibition in experiments for each of these inhibitors one should take into account at least three groups of resistant mutants that differ by both replication capacity and sensitivity to the inhibitor. For example, we considered the known mutants A156T, D168V, and R155Q for inhibitor BILN-2061, mutants A156S, A156T, and T54A for inhibitor VX-950, and mutants A156T, A156S, and T54A for inhibitor SCH 503034. Each of these mutants had a selective advantage over other mutants for a specific concentration of the inhibitor.

It was demonstrated previously that the resistance observed during *in vitro* replicon studies results from the outgrowth of preexisting NS5B mutations rather than from the generation of mutations after the onset of antiviral suppression [8]. Our modeling results are consistent with this evidence: in the whole pool of mutant RNAs the preexisting NS3 protease mutants prevail over mutants generated during the wild-type viral RNA replication in the presence of drug. Moreover the extent of the preexisting mutant contribution depends on the inhibitor concentration, reaching the maximum for the inhibitor concentration range corresponding to the maximal selective advantage of the mutant.

We also use our model to study the efficacy of potential inhibitors targeted to the integral cell factor. These inhibitors can either stop the cell factor formation or reduce its activity in the viral replication complex. Our analysis revealed that the NS3 protease inhibitors are more effective when combined with cell factor inhibitors reducing activity of the viral replication complex.

## Materials and Methods

### Model Description

**Figure 1:**
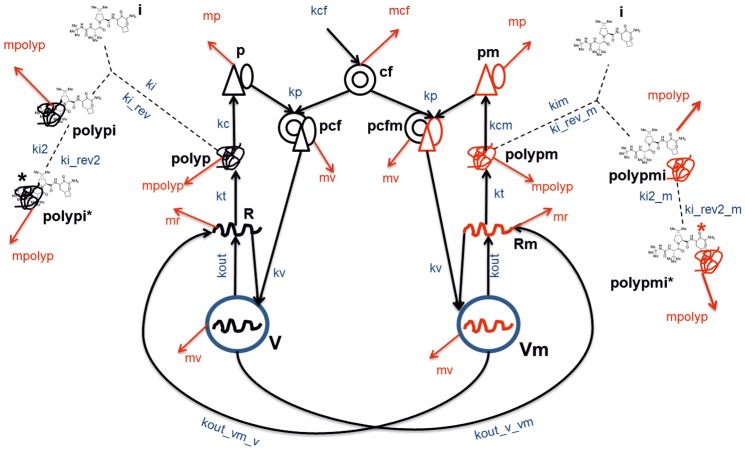
Schematic representation of reactions included in the model. Designations: *V* and *Vm*, vesicles producing wild-type viral RNA and NS3 protease mutant RNA, respectively; *R* and *Rm*, wild-type and mutant viral plus-strand RNAs, respectively; *polyp*, *polypm*, polyproteins translated from wild-type and mutant RNAs, respectively; *p*, *pm* viral nonstructural proteins of the wild and mutant types, respectively; *cf*, cellular factor; *pcf*, *pcfm*, replicase formed by the wild-type viral proteins and by mutants, respectively; *i*, NS3 protease inhibitor; *polypi*, *polypmi*, complex of inhibitor with the wild-type and mutant viral polyprotein, respectively; polypi*, polypmi* covalent complex of inhibitor with the wild-type and mutant viral polyprotein respectively. *kout*, rate constant of the RNAs production by the vesicles *V* and *Vm*, respectively; *kout_v_vm*, mutant RNA production by the wild-type vesicles *V*; *kout_vm_v*, wild-type RNA production by the mutant vesicles *Vm*; *kt*, production for 1000 viral polyproteins; *kc*, *kcm*, production of viral proteins from mutant and wild-type polyproteins, respectively; *kcf*, production of the cellular factor; *kv*, production of vesicles *V* and *Vm*; *kp*, rate constant of the replicase formation by the cellular factor and by the viral proteins; *ki*, *kim*, rate constants of interaction between the inhibitor and the wild-type and mutant polyprotein, respectively; *ki_rev*, *ki_rev_m*, dissociation rate constants for the inhibitor/polyprotein complex for the wild-type and mutant polyprotein, respectively; *ki2, ki2_m* transformation of the noncovalent complex polyprotein/inhibitor to the covalent adduct for wild-type and mutant polyprotein, respectively; *ki_rev2, ki_rev2_m*, transformation of the covalent complex polyprotein/inhibitor to the noncovalent adduct for wild-type and mutant polyprotein, respectively; *mv*, degradation rate constant for vesicles *V* and *Vm*; *mr*, degradation of RNAs; *mpolyp*, degradation of the polyproteins; *mp*, degradation of the nonstructural proteins; *mcf*, degradation of the cellular factor.

We propose the following scheme describing the subgenomic replicon replication in the model (Figure 1). The replicase complexes work according to the *cis*-complementation mechanism, i.e. complexes containing proteins translated from a particular viral genome can mainly replicate it [57], [58]. Therefore, in the model the wild-type (*p*) and mutant (*pm*) viral nonstructural proteins do not mix when forming the replicase complexes *pcf* and *pcfm* with the cellular factor *cf*. For simplicity we assumed the equality of the rate constants *kp* for the interaction of *cf* with either the mutant or wild-type viral proteins. In turn, *pcf* and *pcfm* can bind to wild-type or mutant plus-strand RNA in the cytoplasm. Binding of *pcf* to the wild-type plus-strand RNA *R* results in the formation of vesicles *V* with the rate constant *kv*, whereas binding of *pcfm* to the NS3 protease mutant plus-strand RNA *Rm* results in that of vesicles *Vm* with the same rate constant. In agreement with our previous model [53] we assumed that *cf* restricts the number of active replicases and, hence, of the viral RNA in the cell. Thus, the proposed minimal model is an extension of our previous model, incorporating vesicles and mutants.

Instead of incorporating the rather complicated processes of new RNA strand synthesis inside vesicles explicitly, we assumed that the *V* and *Vm* vesicle*s* produce and release the wild-type *R* and mutant *Rm* plus-strand HCV RNAs into cytoplasm with rate constant *kout*. According to available data, the HCV NS5B polymerase lacks a proofreading function, which results in an estimated erroneous base substitution rate of ∼1 in 10,000 replicated bases [5]. The model takes into account that vesicles *V* can produce the mutant RNAs *Rm* with the rate constant *kout_v_vm*, and, vesicles *Vm* with regard to back mutations can produce the wild-type RNAs *R* with the rate constant *kout_vm_v*. The quantities *R* and *Rm* are translated into wild-type (*polyp*) and mutant (*polypm*) polyproteins, respectively, with the rate constant *kt*.

Based on available information about mechanisms governing the action of known NS3 protease inhibitors, we introduced in the model a reversible process of NS3 protease/inhibitor noncovalent complex formation and a slow transformation process of this complex to the covalent adduct [59], [60]. We assumed that the NS3 protease within the wild-type polyprotein *polyp* can reversibly interact with the NS3 protease inhibitor *inh*, giving rise to the *polypi* complex with the rate constants *ki* and *ki_rev* for the forward and reverse processes, respectively. Similarly to the wild-type polyprotein, the mutant polyprotein *polypm* can interact with the NS3 protease inhibitor *inh* forming the *polypmi* complex with the rate constants *kim* and *ki_rev_m* for the forward and reverse processes, respectively. The transformation of the noncovalent complex to the covalent adduct is modeled in wild-type and mutants with the rate constants *ki2*, *ki_rev2*, and *ki2_m*, *ki_rev2_m*, respectively.

The processing of both wild-type and mutant polyproteins results in the formation of nonstructural proteins, denoted by *p* when cleaved from the *polyp* polyprotein, and *pm* otherwise. The mutant NS3 protease is processed from *polypm* protein. The wild-type and mutant polyproteins can have different processing rates, indeed, differences of the catalytic constant *k_cat_* were reported between the mutant NS3 protease as compared to the wild-type [35], [36], [38]. The processing of *polyp* and *polypm* is described in the model via the rate constants *kc* and *kcm*, respectively. According to the study reported in [61], we assumed that each membranous vesicle *V* or *Vm* with an active replicase complex contains one minus-strand RNA molecule, 6–12 plus-strand RNA molecules, and up to 1000 molecules of each nonstructural protein translated from these plus-strand RNAs. Therefore, *kc* and *kcm* in the model are rate constants of production of 1000 wild-type NS3 proteins and 1000 mutant NS3 proteins, respectively. We note that the wild-type and mutant RNA translation rate constant *kt* also corresponds to synthesis of 1000 polyproteins.

Different NS3 protease mutants have different replication capacity and sensitivity to inhibitors, defining their selective advantage during inhibitor action [11], [12], [38]. In order to account for properties of the potential NS3 protease mutant RNAs, we introduced groups of mutant RNAs *Rm_ij_*, where *i* stands for replication capacity of a group and *j* denotes its sensitivity to inhibitor. Both *i* and *j* can take values from 1 to 3 and so encode low, moderate and high values of the *Rm_ij_* parameter for the group. We assumed that each RNA group replicates in specific vesicles *Vm_ij_*. For example, *Vm_11_* vesicles produce *Rm_11_* mutant RNAs with low replication capacity and sensitivity to inhibitors, *Vm_21_* produce *Rm_21_* RNAs with moderate replication capacity and low sensitivity to inhibitor, and *Vm_32_* produce *Rm_32_* with high replication capacity and moderate susceptibility, etc. The mutant proteins (*polypm_ij_*, *pm_ij_*) and replicases (*pcfm_ij_*) are also specific to the group. Each mutant group is characterized by the set of parameters *kcm_ij_, kim_ij_,* and *ki_revm_ij_*. The parameters *kout_vm_v* and *kout_vm_v*are assumed the same for all mutants. We showed only one mutant group in Fig. 1 for simplicity. The full schematic representation of all biological processes described in the model that takes into account all the mutant groups, is shown in the Supporting Information (Text S1, Fig. S1).

### The Stochastic Method

The stochastic approach is particularly useful for modeling biological processes going on at low concentrations of molecular substances [62]. This is the case with viral RNA under the long-term exposure to protease inhibitors. According to experimental data, the concentration of viral RNA decreases by 2 orders of magnitude as early as 3 days after the action of the NS3 protease inhibitors BILN-2061 (1.75–17.5 μM) and VX-950 (3.5–17.5 μM) [20]. Simulations were performed using the Next Step method of the Gillespie Algorithm, designed specifically for the simulation of coupled chemical reactions [63].

Table S1 gives the propensity functions for the stochastic model of events represented in Fig. 1. The algorithm was implemented in C++ language. Simulations of viral RNA kinetics in the cell were repeated until the standard deviation of the amount of RNA at each time point became less than 0.01 molecules per cell.

Each run of the stochastic model gives an individual dynamics of concentrations of all molecular substances, which differs from run to run. To compare the viral RNA concentrations obtained in the model to the data we averaged the simulation results over 100,000 runs of the model.

### Adjustment of the Model

#### Initial Conditions

Two problems were solved in correspondence with the model: first, the viral RNA should reach a correct stationary state in the absence of inhibitors, and, alternatively, it should be inhibited in the presence of inhibitors according to the data. Action of inhibitors was modeled in two steps. First, the dynamics was calculated in the absence of inhibitors up to the steady state, and then we continued this dynamics after the addition of the inhibitor to the system. Therefore, the initial conditions were fixed at the transfection moment and were identical for the presence of inhibitors as well as for the absence of them. We assumed that initially there are 100 molecules of the wild-type viral RNA *R*, and values of the *V, Rm_ij_, Vm_ij_, polyp, polypm_ij_*, *p, pm_ij_, pcf*, and *pcfm_ij_* variables were set to 0.

#### Parameter Estimation

Values of parameters mv, ki, ki_rev, ki_revm_ij_, ki2, ki_rev2, ki2m_ij_, ki_rev2m_ij_, kout_v_vm, kout_vm_v, kt, mr and kc were determined from the literature data, while values of parameters mp, mpolypkout, kp, kv, kcf, mcf, kim_ij_, and kcm_ij_ were found by the model fitting to the experimental data (Tables 1 and 2).

**Table 1:** Model parameters.

Parameter	Reaction definition	Value	Reference
*kv·*	*V* and *Vm_ij_* formation	3.0 vesicle^−1^ h^−1^	fitting
*mv*	*V* and *Vm_ij_*, pcf, pcfm*_ij_* degradation	0.058 h^−1^	[64]
*kout*	Wild-type RNA (*R*) production by vesicles *V*	1.177 h^−1^	fitting
	Mutant RNA (*Rm*_ij_) production by vesicles *Vm_ij_*	1.177 h^−1^	fitting
*mr*	*R* and *Rm*_ij_ degradation	0.5 h^−1^	[65]
*kt*	Wild-type and mutant polyprotein translation	0.1 h^−1^	[52]
*kc*	Wild-type polyprotein processing	1.0 h^−1^	[66]
*kp*	*pcf*, *pcfm_ij_* formation	0.75 molecule^−1^ h^−1^	fitting
*kcf*	Cellular factor (*cf)* synthesis	5.0 h^−1^	fitting
*mp*	Nonstructural proteins (*p*, *pm*_ij_) degradation	1.0 h^−1^	fitting
*mpolyp*	Mutant (*polypm_ij_*), wild-type (*polyp*) polyprotein degradation,	0.3 h^−1^	fitting
	Complexes (*polypi, polypmi_ij_, polypi* *, *polypmi_ij_* *) degradation	0.3 h^−1^	fitting
*kout_v_vm*	*Rm_ij_* production by vesicles (*V)* containing wild-type viral RNA	2·10^−4^ h^−1^	[5]
*kout_vm_v*	*R* production by vesicles (*Vm_ij_)* containing mutant viral RNA	2·10^−4^ h^−1^	[5]
*ki*	*polypi* formation	0.12 molecule^−1^ h^−1^	[59]
*ki_rev*	*polypi* dissociation	84 h^−1^ *	[59]
		15.3·10^4^ h^−1^ **	[59]
		14.8·10^4^ h^−1^ ***	[59]
*ki2*	Covalent polyprotein/inhibitor complex (*polypi* *, *polypmi_ij_* *) formation	0 h^−1^ *	[59]
		56.52 h^−1^ **	[59]
		59.05 h^−1^ ***	[59]
*ki_rev2*	*polypi, polypmi_ij_* formation from covalent (*polypi* *, *polypmi_ij_* *) complex	0 h^−1^ *	[59]
		0.2952 h^−1^ **	[59]
		0.108 h^−1^ ***	[59]
*mcf*	*cf* degradation	1.0 h^−1^	fitting

*for the polyprotein(wild-type)/BILN-2061 complex.

**for the polyprotein(wild-type)/VX-950 complex.

***for the polyprotein(wild-type)/SCH-503034 complex.

**Table 2:** Model parameters for mutants.

Inhibitor	Mutant	Group	*kcm_ij_* *(h^−1^)	(*ki/ki_ij_*)**	Reference
BILN-2061	A156T	11	0.42	1000.0	[11], [59]
	D168V	22	0.5	100.0	[11], [59]
	R155Q	33	0.95	15.0	[11], [59]
VX-950	A156T	11	0.42	1000	[11], [59]
	A156S	22	0.75	20.0	[11], [59]
	T54A	33	0.95	12.0	[11], [59]
SCH 503034	A156T	11	0.42	100.0	[38], [59]
	A156S	22	0.75	16.0	[38], [59]
	T54A	33	0.95	5.0	[38], [59]

*processing rate constant for mutant polyprotein.

**relation of the rate constant for formation of non-covalent mutant polyprotein*_ij_*/inhibitor complex to the rate constant for formation of non-covalent polyprotein (wild-type)/inhibitor complex.

#### Parameter estimation based on literature data

The initial estimates of *mv* and *mp*, the degradation rate constants for vesicles and the viral complexes of nonstructural proteins correspondingly were taken from literature. These initial estimates were used to determine a range for the parameter values inside which the model adjustment was performed. The half lives for nonstructural proteins were determined to be within the 10–16 h interval [64]. On this basis the initial *mv* value was set to 0.1 h^−1^. The *mv* value was corrected during model adjustment and was computed as 0.058 h^−1^. Knowing HCV RNA half-life in the cytoplasm (2.3 hours) [65], *mr* was set to 0.5 h^−1^. On the basis of the duration of the rate-limiting step of processing of polyprotein (wild-type) [66], the rate constant for processing of the polyprotein (wild-type) *kc* was set to 1 h^−1^. The rate constant of translation of 1000 molecules of polyprotein *kt* was set to 0.1 h^−1^ [52].

In order to describe the interaction of inhibitors BILN-2061, VX-950, and SCH 503034 with the wild-type polyprotein, values for constants *ki*, *ki_rev*, *ki2*, and *ki_rev2*, characterizing the rates of the two stages of covalent adduct formation, were taken from [59]. Values of similar constants for inhibitor ITMN-191 were taken from [60]. For inhibitor SCH 900518, the corresponding constants were estimated from the half life time (2 hours) of the covalent NS3/inhibitor complex and the overall inhibition constant K_i_* [25].

For the interaction of mutant polyprotein with inhibitors BILN-2061, VX-950, and SCH 503034, the formation rate constant *kim_ij_* for the non-covalent mutant polyprotein/inhibitor complex was estimated via the ratio of *ki* to the relative sensitivity of mutant *ij* to the inhibitor (Table 2) Rate constant *ki_rev2_m_ij_* for mutant polyprotein/inhibitor complex dissociation was the same as for wild polyprotein/inhibitor complex *ki_rev2*. The rate constant *ki2_m_ij_* of covalent mutant polyprotein/inhibitor adduct formation from the non-covalent mutant polyprotein/inhibitor complex was assumed equal to *ki2*, and the rate constant *ki_rev2_m_ij_* of the reversed process was taken equal to *ki_rev2*.

We estimated the rate constant *kout_v_vm_ij_* of the mutant RNA *Rm_ij_* production by vesicles *V* as *kout_v_vm_ij_*  = 2×10^−4^ h^−1^ using the estimation of the probability of one nucleotide substitution during the HCV genome replication (from 10^−3^ to 10^−5^ mismatches per nucleotide copied [5]). We also assumed that the rate constant *kout_vm_v* of the wild-type RNA production by the mutant vesicles *Vm_ij_*is equal to *kout_v_vm*.

The paper [11] contains the most complete information about replication capacity (RC) of the known NS3 protease mutant replicons and their susceptibility to inhibitors BILN-2061 and VX-950. Based on this information the initial estimates were obtained for parameters *kim_ij_* and *kcm_ij_* for the mutants resistant to BILN-2061 and VX-950. These estimates were later varied in a bounded range of values during model fitting to experimental data. Values of parameters *kim_ij_* for SCH 503034 resistant mutants were assigned based on experimental data on their susceptibility to this inhibitor published in [38]. The experimental data on mutant susceptibility used for the model adaptation are shown in Table 3.

**Table 3:** Data on resistance of the NS3 protease mutants to the BILN-2061, VX-950 and SCH 503034 inhibitors used for the model adaptation.

Mutant	Relative RC level without an inhibitor (%)	Relative BILN-2061 susceptibility fold [11]	Relative VX-950 susceptibility fold [11]	Relative SCH 503034 susceptibility fold [38]
Wild-type	100	1.0	1.0	-
V36M	43.71	0.8	0.7	-
T54A^+++, ###^	66.75	0.6	6.7	6
R155Q***	74.51	29.6	1.3	-
R155K	99.19	147.5	5.4	-
V36M/R155K	75.94	139.9	3.3	-
A156T* ^,+, #^	36.03	366.6	96.3	80
A156S^++,##^	52.93	2.8	19.0	8
A156V	0.6	445.0	63.1	-
D168V**	42.95	193.6	0.2	-
D168A	5.34	63.3	0.1	-

*, **, ***mutants resistant to BILN and assigned to the groups with low, moderate, and high replication capacity and susceptibility to the inhibitor.

+, ++, +++ mutants resistant to VX-950 and assigned to the groups with low, moderate, and high replication capacity and susceptibility to the inhibitor.

#, ##, ### mutants resistant to SCH 503034 and assigned to the groups with low, moderate, and high replication capacity and susceptibility to the inhibitor.

The data for BILN-2061, VX-950, and SCH 503034 inhibitors from Table 3 were used to classify all mutants in three groups.

The group with both *low* relative replication capacity and relative susceptibility to these three inhibitors was associated with mutant A156T.

The group with both *moderate* relative replication capacity and relative susceptibility to an inhibitor was associated with mutant D168V for BILN-2061 and mutant A156S for SCH 503034 and VX-950.

The group with both *high* relative replication capacity and relative susceptibility was associated with mutant R155Q for BILN-2061 and with mutant T54A for SCH 503034 and VX-950.

Mutants A156V and D168A were not considered, since they almost do not replicate. In contrast to other mutants, V36M/R155K and R155K require more than one nucleotide substitution. As such mutants arise rarely [5] we did not take them into consideration. For BILN-2061 and VX-950, we also omitted the V36M mutant, since it has very high susceptibility to both inhibitors, comparable to that of the wild-type.

#### Parameter estimation by model fitting to experimental data

The unknown parameters are to be found as a solution to an inverse problem of fitting the computed model output to experimental data. The parameter optimization and sensitivity studies for the stochastic model are computationally expensive, as they require multiple evaluation of the model to collect sufficient statistics. In order to facilitate the calculations, we optimized some of the parameter values and investigated sensitivity for the deterministic version of the model in the form of ordinary differential equations (see Text S1). We showed previously that the deterministic model elaborated in [53] correctly simulates both the viral RNA dynamics to the steady state in the absence of inhibitors and the RNA inhibition kinetics under the action of inhibitors when the RNA concentration remains relatively large in the cell. For small RNA concentrations, the deterministic model did not reproduce the experimental RNA dynamics. As expected, the new deterministic model is able to correctly describe only the first phase of the two-phase RNA inhibition kinetics in the presence of drug (Text S1, Fig. S2, S3). According to the experimental data that we used for the model analysis, the viral RNA concentration was essentially smaller during the second phase (in approximately four days since the cells were treated with various inhibitors) than in the first phase and varied in a narrow range of values. We assumed that this property of the RNA dynamics should not influence the model sensitivity significantly. Therefore, we analyzed the deterministic model only in the time interval when the model correctly simulates the experimental viral RNA dynamics.

In this paper we used the enhanced Differential Evolution Entirely Parallel method. The original DEEP [67] method was successfully applied to the problem of the regulatory gene network reconstruction [68]. Details are given in Text S4. We used the optimization procedure to determine values of 18 model parameters in total (7 free parameters for the deterministic model and 11 mutant-related parameters for the stochastic model), namely *kp*, *kv*, *mp*, *mpolyp*, *kout_v*, *kcf*, *mcf*, *kim_ij_* (i = j, i = {1, 2, 3}), *kcm_ij_* (i = j, i = {1, 2, 3}) for considered mutant groups resistant to BILN-2061 and VX-950. Therefore, the deterministic model has 11 parameters; 7 of these parameters were optimized and 4 were estimated from literature. The stochastic model has 22 parameters, including 11 parameters from the deterministic model and 11 mutant-related parameters. For the stochastic model, only mutant-related parameters were optimized, while all the rest parameter values were taken from the optimization results in the deterministic model. Thus, totally 18 parameter values were optimized, including 7 parameters from the deterministic model and 11 mutant-related parameters from the stochastic model. The data consisted of 13 experimental observations of the viral RNA concentration after 3 days of treatment with different doses of BILN-2061 and VX-950 [35] and time course data of viral RNA inhibition by the same inhibitors [20].

The objective function is determined as the sum of squared differences between the model output and data. We considered one vesicle as one active replicase, so that the stationary concentration of vesicles was defined as the stationary number of active replicases. According to calculations in [69] the average number of replicases in one cell is approximately equal to one hundred. Consequently, we also used two additional criteria to penalize deviation of the model from a desired behavior. The first constraint postulates that the RNA concentration should reach the stationary value in 48 hours, and the second one defines the stationary number of vesicles equal to 100. Ten sets of the optimal parameter values for *kp*, *kv*, *mp*, *mpolyp*, *kout_v*, *kcf*, *mcf* and 6 sets for *kim_ij_*, *kcm_ij_* were selected as the best results of the fitting (see Text S4). We have applied the bootstrap method using 1000 replicas to obtain the lower and upper limits of all optimized parameters (See tables in Text S4).

#### Sensitivity analysis

We investigated sensitivity of the deterministic model to perturbed values for all 11 parameters in the model (*kp*, *kv*, *mp*, *mpolyp*, *kout_v*, *mr*, *kt*, *kproc*, *mv*, *kcf*, *mcf*) and for parameter *Rinit* defining the initial number of virus RNA molecules. First, we calculated values of the objective functional including all penalizing terms (‘full cost’) when each parameter was varied around its optimal value with all the rest parameters being fixed at the optimal values. We allowed the full cost function to increase up to 150% of its optimal value and determined corresponding variation ranges for all parameters. Five parameters (*kout_v*, *mr*,*kt*, *kproc*, and *mpolyp*) had these ranges equal to 15–35% of their optimal values, while these ranges were more than 50% for all other parameters (see resulting figures in Text S2). This analysis was performed for various sets of the optimal parameter values found by fitting, showing similar results for different sets (Text S2).

We also studied the sensitivity by investigating how the model behaves when all parameter values are perturbed simultaneously. A method (Text S3) was applied to find a vicinity of the optimal parameter values in the form of a multi-dimensional box in the parameter space such that about 80% of its volume correspond to model solutions whose cost deviates from the optimal cost value less than a predefined quantity (see resulting figures in Text S2). The cost was calculated as the logarithm of the objective functional penalized by the constraint associated with the stationary number of vesicles. The use of logarithm was motivated by the fact that all data implemented in the fitting procedure was in the logarithmic scale. In this computational experiment, most of the parameters demonstrated large variation ranges corresponding to allowed variation range of the cost. The most sensitive parameters were *kt*, *kproc*, and *mv* with the variation ranges of not more than 10% of their optimal values.

## Results

### 1. Modeling of the steady-state attainment of viral RNA concentration in the absence of inhibitors

In the model we consider viral RNA both explicitly in the cytoplasm and also implicitly inside the vesicles. To calculate the amount of viral RNA inside the vesicles, we assumed a ratio of 6 positive strand RNA molecules per vesicle, and a single negative strand RNA molecule inside each vesicle, estimated from an average over all vesicles [61].

**Figure 2:**
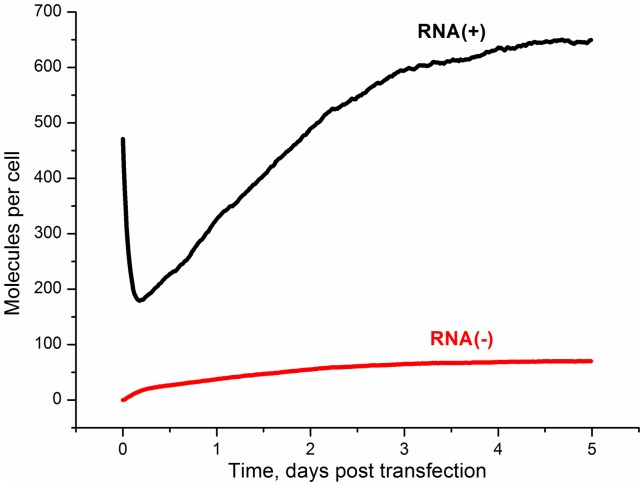
The kinetics of the viral RNA production in a cell after the subgenomic HCV replicon transfection in the absence of the NS3 protease inhibitors. Black line: plus-strand RNA; red line: minus-strand RNA.

The calculated kinetics of plus- and minus- strands of the viral RNA (the sum of the amounts of RNA in cytoplasm and vesicles) after the subgenomic HCV replicon transfection are shown in Fig. 2. Recall that each type of RNA strand is represented by both wild-type and mutant RNA species. As it can be seen from the figure, the steady-state RNA number was reached in 3–4 days; the number of the viral plus- and minus-strand RNAs was 678 and 71 molecules/cell, respectively. The calculated amounts of viral plus- and minus-strand RNAs and time, when steady-state was reached, are in good agreement with experimental data. In [61] this time was estimated as 2 or 3 days post-transfection, stationary concentrations of plus- and minus- RNA strands were approximately 1000 and 100 molecules per cell, respectively.

One feature of the plus-strand viral RNA kinetics at initial time is worthy of special mention. According to Fig. 2 the amount of plus-strand viral RNA in the cell decreases during several hours just after transfection. This can be explained by the delay in production of viral RNA that is caused by the time necessary to synthesize the viral proteins responsible for the viral RNA replication. Immediately after transfection only the plus-strand RNA is available in the cell, and its degradation causes the decrease in the plus-strand RNA amount. Similar behavior was also observed in experiments [70].

### 2. Kinetics of the NS3 protease mutant RNA production in the absence of inhibitors

The HCV RNA-dependent RNA polymerase NS5B lacks a proofreading function [5], therefore here we have assumed that drug-resistant mutant viral RNAs preexist in a treatment-naive replicon population [8], [12]. The data on replication capacity and sensitivity to inhibitors are available for many mutants [11], [12], [25], [38]. Here we consider the most important and well known NS3 protease mutants that confer resistance to the BILN-2061, VX-950 and SCH503034 inhibitors (Table 3).

**Figure 3:**
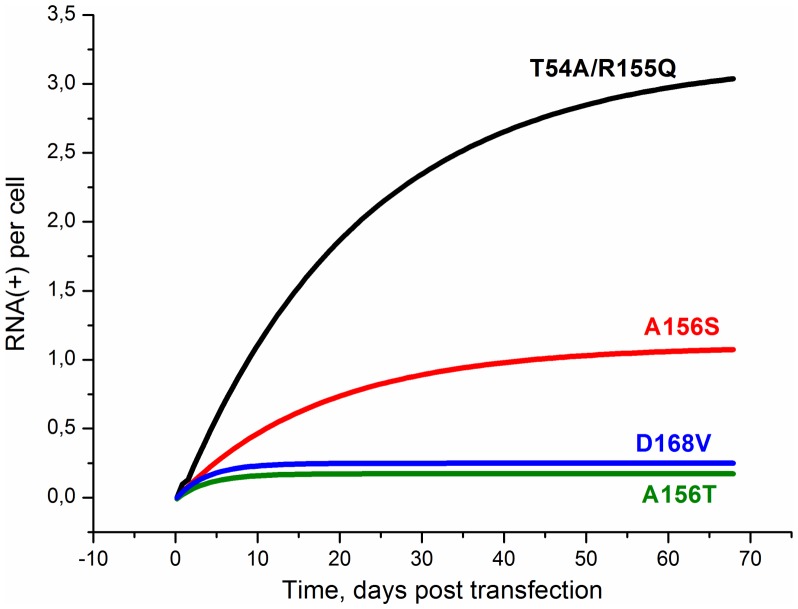
The kinetics of the mutant plus-strand RNA production in the absence of inhibitors after transfection with wild-type replicon. The kinetics were calculated for mutants A156T, D168V, A156S, T54A and R155Q with rate constant for polyprotein processing *kcm_ij_* (see Table 2).

The dynamics of production of the NS3 protease mutant plus-strand RNAs after transfection with wild-type replicon in the absence of inhibitors is shown in Fig. 3. It is clearly seen that in our model mutants differ both in steady-state concentration and time necessary to reach the steady-state. These differences are easily explained by different replication capacity of mutants, defined in the model by the rate constant for processing of the polyprotein (*kcm_ij_*). One can see in Fig.3 that the stationary concentration of a mutant increases as this constant rises. Therefore R155Q and T54A that have the highest replication capacity are the most abundant mutants in the viral RNA pool. It is worth to note that mutants with higher replication capacity need more time to reach the steady-state.

The steady-state concentrations of viral RNAs for all the mutants are given in Table 4. Note that the time to reach the steady-state grows as the rate constant of polyprotein processing increases. For example, the time to reach the steady state for R155Q (*kcm* 0.95 h^−1^) and T54A (*kcm* 0.95 h^−1^) was about two months, and for A156T (*kcm* 0.42 h^−1^) this time was only a few days. The total fraction of all drug resistant mutants in the resultant RNA pool at day 60 post-transfection equals about 1% in our model that is close to the experimental estimate of the fraction of the preexisting drug resistant mutant RNAs for the same time period, that is approximately 1% [8], [12].

**Table 4:** Stationary concentration of the viral RNA calculated by the model.

HCV replicon	Rate constant for polyprotein processing *kcm_ij_* (h-1)	Minus-strand RNA (molecules per cell)	Plus-strand RNA (molecules per cell)	Time to reach steady state (days)
WT	1	70	670	5
A156T	0.42	0.018	0.17	10
D168V	0.5	0.025	0.23	10
A156S	0.75	0.12	1.15	40
T54A	0.95	0.32	3.06	60
R155Q	0.95	0.32	3.06	60

### 3. Suppression of viral RNA in the presence of the NS3 protease inhibitors

**Figure 4:**
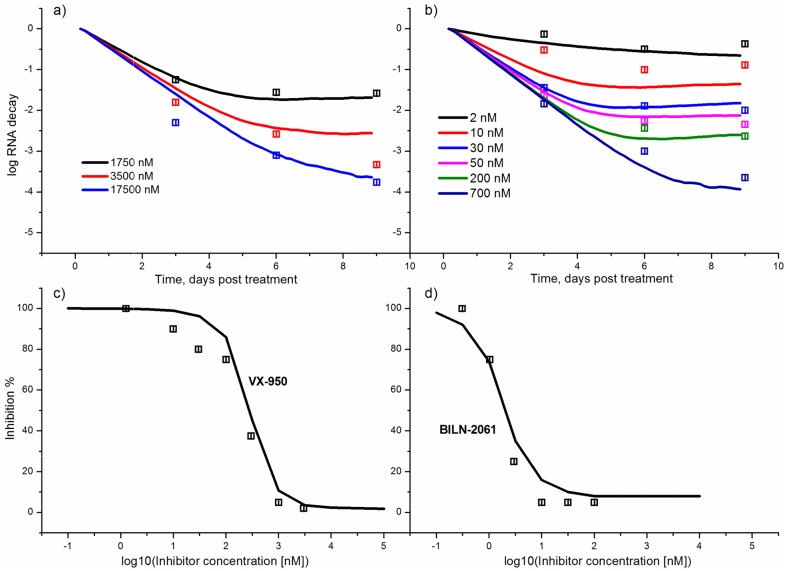
Kinetics of the viral RNA suppression at different concentrations of the NS3 protease inhibitors. (A) the VX-950 inhibitor, (B) the BILN-2061 inhibitor. The insert shows the concentrations of the inhibitors used. The dependence of the viral RNA suppression on the increasing concentration of the VX-950 (C) and BILN-2061 (D) inhibitors is shown for the second and third day post-treatment, respectively. The level of suppression was calculated as the ratio of the RNA for the given inhibitor concentration to the steady-state RNA level. A solid line is used to show the model output, points correspond to the experimental data [20], [35].

The inhibitor action is modeled after the system reaches the steady-state. The experimental kinetics of the viral RNA suppression in the presence of the BILN-2061 (Fig. 4a) and VX-950 (Fig. 4b) inhibitors [20] were used for fitting together with the dependencies of the suppression rate on inhibitor concentration (Fig. 4c,d) [35]. As it is clearly seen, the model correctly reproduces experimental data.

**Figure 5:**
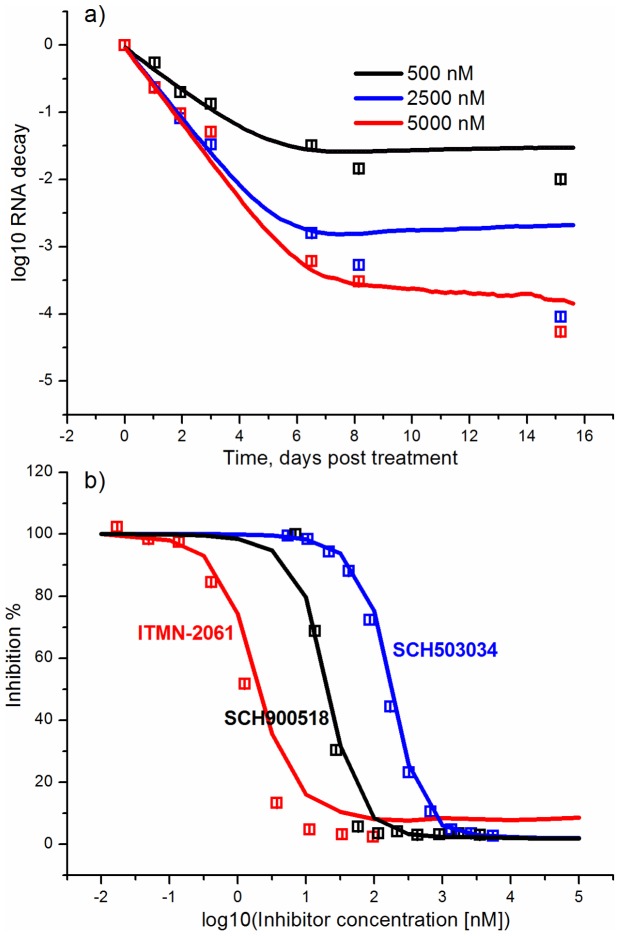
The predicted kinetics and viral RNA suppression rate as compared with data not used for parameter estimation. (a) Kinetics of the viral RNA suppression by different concentrations of the SCH503034 inhibitor; (b) The dependencies of the viral RNA suppression rate on the increasing concentration of the SCH-503034, SCH-900518 (3-d day post treatment) and ITMN-191 (2-d day post treatment) inhibitors. Solid line is used to display the model output and points correspond to the experimental data [22], [25], [29].

**Figure 6:**
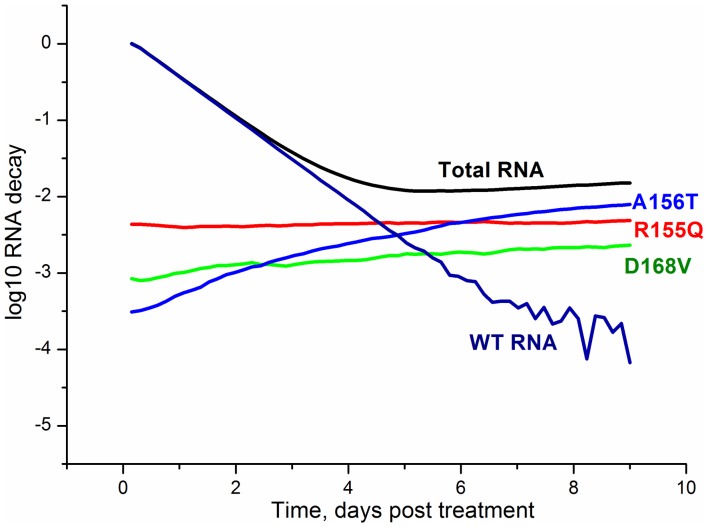
The dynamics of the wild-type and mutant viral RNAs in the presence of the BILN-2061 inhibitor. The data are presented for the A156T, R155Q and D168V mutants, wild-type and total viral RNA. The concentration of inhibitor is 30 nM.

The predictive power of the model was examined using the experimental kinetics of the viral RNA suppression with the SCH-503034 inhibitor (Fig. 5a) [22], and the experimental data on dependencies of the viral RNA suppression rate on the increasing concentration of the SCH-503034 [22], SCH-900518 [25] and ITMN-2061 [29] inhibitors (Fig. 5b). These data were not used for parameter estimation. The modeling results are in good agreement with data, confirming the model's ability to predict the kinetics of viral RNA production under exposure to protease inhibitors. However, it should be noted that the model output visibly deviates from the experimental data at late times especially at time point that corresponds to 15 days post-treatment with 2500 nM of the SCH503034 inhibitor. This deviation may be explained by low concentration of viral RNA due to inhibitor action or low sensitivity of the experimental measurement methods, as well as by the fact that the model does not account for the possible activation of IFN synthesis during the action of NS3 inhibitors [33], [71], [72]. It is known that HCV NS3 protease is able to suppress IFN-I production [73], [74]. However, currently there is little agreement about the level of viral protein inhibition at that IFN starts to play an important role in the viral RNA suppression. According to [72,] the NS3 protease inhibitor concentration over 100 EC50 is needed to activate the IFN synthesis. This level exceeds that used to calculate the kinetics in Fig.5a.Interestingly, the characteristic two-phase behavior of the viral RNA kinetics during the treatment with the BILN-2061 (Fig. 4a), VX-950 (Fig. 4b) and SCH503034 (Fig. 5a) inhibitors, that consists of the rapid decrease in the first phase and the slow decrease of the viral RNA concentration in the second one, is explained in our model by emergence and selection of drug resistant mutants. Indeed, as is shown in Fig. 6, which presents the model output for each drug resistant mutant in the presence of the BILN-2061 inhibitor at 30 nM concentration, the wild-type concentration decreases over time, while the mutant concentration increases, reaching a plateau, that slows down the total decrease of the viral RNA amount. The concentration of drug resistant mutants exceeds that of the wild-type after 5 days of treatment.

The experimental dependencies of the viral RNA suppression rate shown in Fig. 4c and Fig. 4d were obtained after two days of treatment with the BILN-2061 [35] and three days of treatment with VX-950 [20] inhibitors respectively. According to the model, after such a short period of treatment with inhibitors, the viral RNA is mostly represented by wild-type RNA and drug resistant mutants had no influence on observations because of low concentration (Fig. 6).

Consequently, the dependencies of the viral RNA suppression rate on the increasing concentration of the SCH-503034, SCH-900518 and ITMN-191 inhibitors (Fig. 5b) were calculated without taking mutants into account as their contribution at time when the effect of inhibitor action was measured (at day 3 from the start of treatment for SCH-503034 and SCH900518 and day 2 for ITMN-191) is negligible according to our model. This also explains why our previous model [53], in which the existence of drug resistant mutants was not considered, nevertheless reproduced well the system dynamics at short time intervals of inhibitor action. Note that according to the numerical results as early as after the fifth day of treatment with inhibitors the concentration of the resistant mutant RNA prevailed over the wild type. This is in agreement with the qualitative literature data. For example, McCown and co-authors [75] showed that resistant replicons appeared in the cell culture as early as after the third day of treatment with the NS3 protease inhibitor VX-950. The number of different mutants identified in experiment increased with time of treatment. In [76] it was shown that the concentration of the NS5B resistant mutant significantly increased on the fourth day of treatment of replicon cells with the NS5B polymerase inhibitor. It is also known that in the majority of patients resistant mutants are found after 2–4 days of monotherapy with the NS3 protease inhibitors GS-9256 and GS-9451 [77].

### 4. Selection of the NS3 protease mutant RNAs in the presence of the BILN-2061 and VX-950 inhibitors

The ability of viruses to develop resistance to drugs is one of the central problems in pharmacology. The selection of drug-resistance mutants under inhibitor pressure depends on many factors, including the concentration of the inhibitor in the cell. For example, in [12] 454 deep sequencing was used to assess the dynamics of mutants emerging *in vitro* under various selective pressures with TMC380765, a potent macrocyclic HCV NS3/4A protease inhibitor. It was shown that depending on the concentration of TMC380765, distinct mutational patterns emerged. Besides, the analysis of the selective advantage and replication capacity of the known NS3 protease mutants was carried out at various concentrations of the BILN-2061 and VX-950 inhibitors [11], [13]. Importantly, that in contrast to data from [12] these investigations used transient-replication assays, but not the wild-type replicon. We use our model to investigate the selection of mutants resistant to the BILN-2061 and VX-950 inhibitors and to study their patterns in the cells transfected with the wild-type replicon.

**Figure 7:**
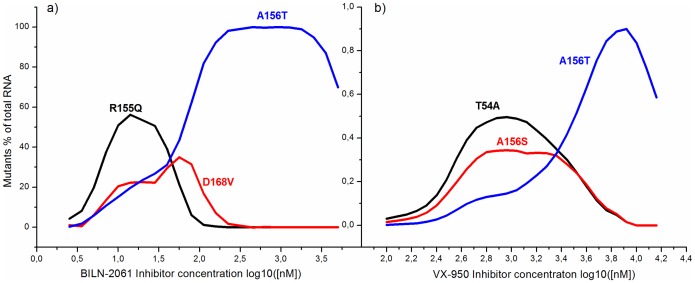
The abundance of mutants in the cell vs. the increasing concentration of BILN-2061 (a) and VX-950 (b) inhibitors.

In Fig. 7 the predicted dependence of mutant abundance in the cell vs. the increasing concentration of the BILN-2061 (a) and VX-950 (b) inhibitors is shown after 9 days of treatment. This parameter was calculated as the ratio of the amount of the mutant RNA of one type to the whole amount of RNA including mutants and wild-type.

According to the figure, our model suggests the the maximum abundance of each mutant type is observed at different concentration of the inhibitor. The peaks of abundance curves for mutants with higher susceptibility to inhibitor are usually shifted towards lower inhibitor concentrations. For example, in case of the BILN-2061 inhibitor (Fig. 7a) the first, second and third peaks correspond to the R155Q, D168V and A156T mutants, that show high, median and low sensitivity to the inhibitor, respectively. For VX-950 inhibitor (Fig. 7b) the abundance peaks for the T54A and A156S mutants almost coincide. The next peak, which belongs to the A156T mutant, forms at higher concentration of the inhibitor. Note that the A156T mutant, which has the lowest replication capacity in comparison to wild-type in the absence of inhibitors and at the same time the lowest susceptibility to the BILN-2061 and VX-950 inhibitors, dominated over other mutants at high concentrations of inhibitor in almost all cases. Consequently, for each mutant it is possible to theoretically propose the ranges of inhibitor concentrations (corresponding to peaks in Fig. 7), over which this mutant is predominantly selected, i.e has selective advantage. The model showed that at low inhibitor concentrations the selective advantage of a mutant is not so strongly pronounced, and several different mutants can be selected simultaneously.

The obtained results are in agreement with experimental data on relative replication capacity and selective advantage of the NS3 protease mutants resistant to the BILN-2061 and VX-950 inhibitors [11]. Particularly the calculated positions of the abundance curve peaks for mutants in the model are in agreement with ranges of inhibitor concentrations over which the peaks of selective advantage for these mutants occur [11]. In addition, as Fig.7 shows, our model permits each inhibitor to have the concentration ranges over which several different mutants can be effectively selected simultaneously. This result agrees with the protease mutants' fitness profiles obtained in [11].

The curves of mutant abundance at different inhibitor concentrations allow us to draw the hypothesis on how the viral RNA pool for each kinetic curve in Fig. 4 is composed. For example, at high concentration of BILN-2061 inhibitor (700 nM) and day 9 post-treatment the viral RNA is dominated by the A156T mutant (relative amount 90%) and for 50 nM concentration of this inhibitor the RNA pool is mainly composed of the R155Q and D168V mutants with frequencies 43% and 28% of the population, respectively.

### 5. The impact of the preexisting and newly emerging mutants on the selection of drug resistant viral RNAs in the presence of inhibitor

The impact of preexisting and newly emerging under drug pressure mutants in the drug resistance acquisition by the HCV replicon is widely discussed in the literature [8], [12]. Recently Robinson et al. (2011) [8] used the fluorescent protein-labeled genotype 1 HCV replicon cell lines to monitor the kinetics of resistance emergence and demonstrated that resistance observed during *in vitro* replicon studies results from the outgrowth of preexisting mutations rather than from the generation of new mutations after the onset of antiviral suppression. Significantly extending the limit of detection as compared with previous approaches [12] this study, however, did not shed light on the composition of the HCV replicon population within a single cell. It is hardly possible to do such measurements with current experimental techniques and, therefore for the present, *in silico* modeling provides the only tool to address this question. We use our model to assess the relative frequency of preexisting and newly emerging drug-resistance mutations within a single cell and compare our estimates with those obtained by Robinson et al. (2011) [8] in replicon cell lines.

We estimate the contribution of preexisting and newly emerging drug-resistance mutants into total RNA pool as the ratio of the amount of mutants of each type to the whole viral RNA at the 9th day post-treatment. As preexisting mutants we consider all the mutants descending directly from mutants that existed at steady-state before the treatment starts. The newly emerged mutants are mutants that arise during the wild-type viral RNA replication under drug treatment, as well as their offspring.

**Figure 8:**
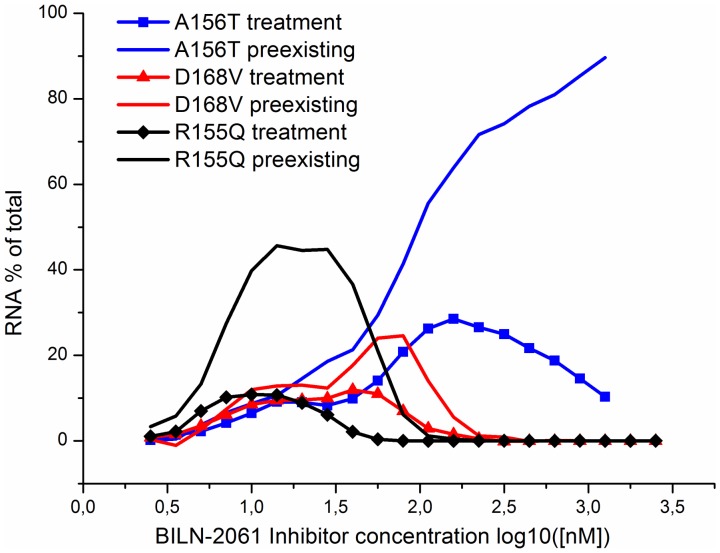
The fraction of preexisting and newly emerging mutants in total RNA pool at different concentrations of BILN-2061 and at day 9 post-treatment. Calculations were done for R155Q (red), D168V (blue) and A156T (black). Curves for preexisting mutants are shown with solid lines, dashed lines are used to display curves for newly emerging mutants.

Fig. 8 illustrates how the predicted contribution of the preexisting and newly emerging R155Q, D168V, A156T mutants into the total RNA pool changes with the increase of the BILN-2061 inhibitor concentration. It is clearly seen that the offspring of the preexisting mutants is presented in a greater amount in comparison to newly emerged mutants. The differences in fractions of the preexisting and newly emerged mutants appeared to be the most obvious in the ranges of inhibitor concentrations that correspond to the maximal selective advantage of each of the considered mutants (see Fig. 7 and 8). This model based hypothesis is in a good agreement with the widely accepted opinion that the preexisting mutants contribute decisively to the drug resistance acquisition by the HCV replicon [8], [12].

### 6. The analysis of the combined action of NS3 protease inhibitors and cell factor inhibitors

Due to the low fidelity of the polymerase NS5B and the high rate of turnover of the viral RNA, drug resistance is and will continue to be a major problem in the development of specific HCV inhibitors. The use of combinations of antiviral compounds that have different targets could be effective in preventing the emergence of drug-resistant viruses. In literature the effects of the combined action of the NS3 protease inhibitors and inhibitors of host factors such as cyclophilins that are also essential for viral replication are described [14], [78].

**Figure 9:**
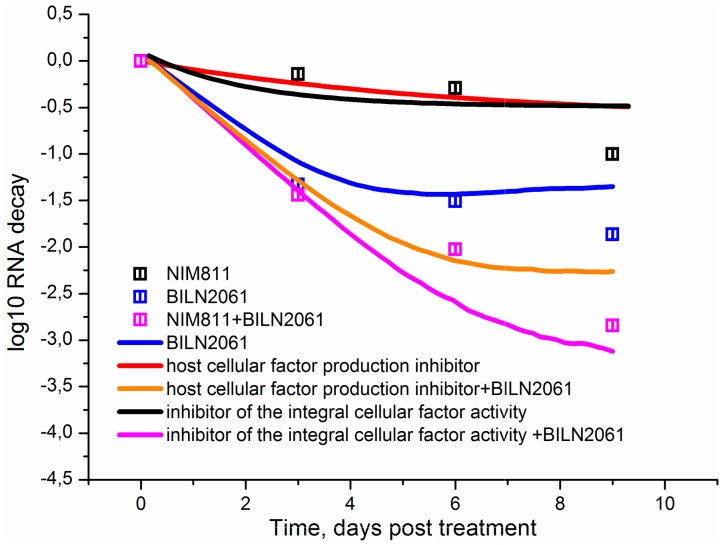
The kinetics of the viral RNA suppression in the presence of the combination of the inhibitor of the integral host cellular factor production and BILN-2061 and the combination of the inhibitor of the integral host cellular factor activity and BILN-2061. Solid lines correspond to the model output. Experimental data [14] are shown with points.

The strong potency of the cyclophilin analogues against HCV indicates that the host cellular factors essential for viral replication can be envisaged as effective antiviral agents. Compounds targeting cyclophilin, for example NIM811 [14], exhibit their action by blocking activity of the host factor essential for virus replication, however theoretically the other mechanism for targeting the host factor can be considered, namely the inhibition of the host cellular factor production. While such inhibitors have not been developed yet, we can use our model to test their utility as potential targets for antiviral therapy and compare the strength of the viral RNA suppression by the combination of the NS3 protease inhibitor with either the host cellular factor activity inhibitor or the host cellular factor production inhibitor. It should be noted that in the model an integral cellular factor stands for both known [18], [19] and unknown host proteins involved in the formation of active replicase. To model the action of the integral cellular factor inhibitor decreasing the activity of the viral replicase complex we vary the parameter *kout*, while the action of an inhibitor that blocks the cellular factor production was modeled by variation of the parameter *kcf*. In order to compare these effects the values of the parameters *kout* and *kcf* were set in a way that the levels of the viral RNA suppression in the case of the individual action of each inhibitor coincide (Fig. 9).

It is clear from Fig. 9 that the calculated kinetics of the viral RNA suppression in the presence of BILN-2061 in combination with the inhibitor of the integral cellular factor activity is in good agreement with the experimental kinetics of the HCV RNA reduction in the presence of BILN-2061 and NIM811 [14]. The model suggests that BILN-2061 is less effective when combined with the potential inhibitor of the cellular factor production than with the inhibitor of the integral cellular factor activity.

## Discussion

Despite many new antiviral drugs appeared recently, the effective treatment of severe chronic diseases caused by such viruses as HCV, human immunodeficiency virus (HIV), and hepatitis B virus (HBV) is complicated by drug-resistant variants of these viruses. The HCV RNA-dependent RNA polymerase, as well as the HIV and HBV reverse transcriptases, are intrinsically error-prone and have no proofreading function. These properties of the virus enzymes promote the appearance of multiple genetic variants of the viruses (quasispecies) that co-exist with the dominating wild-type virus. These quasispecies may contain mutants much less sensitive to antiviral drugs. In the absence of drugs, the drug-resistant variants appear rarely in patients, but the antiviral drug treatment leads to fast selection of the drug resistant mutants [79], [80].

The subgenomic HCV replicon cell culture systems are widely used in studies of the HCV drug resistance. These systems are applied for selection of the drug-resistant replicon clones in the presence of drugs and subsequent sequencing of viral genes in order to determine mutations and investigate replication capacity of mutants, as well as their sensitivity to inhibitors [11], [12], [25]; [38]. The selective advantage profiles were obtained for the panel of HCV mutant replicons in the presence of the NS3 protease inhibitors BILN-2061, ITMN-191, and VX-950. This study revealed the dominant mutants that exhibit the high levels of selective advantage in different drug concentration ranges. It was found that five mutants (R155K/Q, A156T, and D168A/V) showed significant levels of selective advantage after treatment with >10 nM of BILN-2061 Mutant R155K dominates under ITMN-191 treatment in a wide concentration ranges for this inhibitor. The VX-950 treatment at concentrations more than 3 μM leads to selective advantage of mutants A156T and A156S [11]. The mutants resistant to the NS3 protease inhibitors SCH503034, SCH 900518 and TMC380765 were also identified and characterized in details [12], [25], [38].

Many cellular proteins are known at the moment that participate in HCV replication in hepatoma cells [19], [81], as well as cellular proteins associated with the HCV plus-strand RNA [82]. It is well known that viral proteins are able to reprogram cell metabolism and bioenergetics [83]. Our model reported in [53] allowed us to describe the kinetics of the viral RNA suppression in the presence of both integral cellular factor and viral protein inhibitors on short time intervals (up to 3 days). In the model by Binder et al. [54], the replication processes of HCV replicon were considered in more details as compared to the study [53]. However, the inclusion of the drug action into that model also does not allow to correctly describe the long-term effect of the drugs (Fig. S3).

Here we present a minimalistic stochastic model of subgenomic HCV replicon replication in Huh-7 cells which takes into account the existence of drug-resistant mutants. The constructed model is based on the previous modeling attempts [53], [54]. For simplicity, we did not consider the details of viral RNA replication in vesicles. The model also includes only the generalized cell factor like in [53].

This model reproduces well the viral RNA kinetics in the cell from the moment of the subgenomic HCV replicon transfection until steady state, as well as the viral RNA suppression kinetics in the presence of NS3 protease inhibitors. The model was fitted to the experimental kinetics of viral RNA suppression in the presence of inhibitors BILN-2061 and VX-950 [20]. The model predictions were successfully tested on data on the kinetics of viral RNA suppression by inhibitor SCH503034 [22], as well as on data on the dependence of the viral RNA suppression rates on concentrations of SCH-503034, SCH-900518, and ITMN-2061 inhibitors [22], [25], [29]. These data were not used for the model fitting.

In contrast to our previous deterministic model, the suggested here stochastic model describes the two-phase dynamics of viral RNA suppression on prolonged time intervals of inhibitor action. This fact deserves special attention. The deterministic model developed earlier was extended to incorporate the emergence and selection of drug-resistant mutants. The extended deterministic model as well as the stochastic one was able to correctly reproduce the establishment of the viral RNA steady-state after transfection in the absence of inhibitors (Text S1, Fig. S2 (a)). However, the experimentally observed dynamics of the viral RNA suppression by the action of inhibitors could be described by the deterministic model only during the short period of time analogously to our previous model [53]. In contrast, the stochastic model solutions are in a good agreement with experimental data (Text S1, Fig. S2 (b)). Consequently, both the stochastic nature of the processes in the cell for small concentrations of viral RNAs and emergence and selection of resistant mutants prove to be key factors underlying the adequate description of the long-term kinetics.

Note that two-phase kinetics of RNA suppression are also observed during the treatment of replicon cells with different concentrations of IFN-α. In [84] the kinetics of viral RNA suppression with different IFN-α concentrations were described by the simple deterministic model that considered both the production and degradation rates of viral RNA under IFN-α action. In contrast to Dahari's model [84], the biphasic decline of viral RNA under the inhibitor action arises in our model due to formation of resistant mutants. It should be noted, that the resistance can also occur in the case of antiviral IFN action. In particular, the literature reports on the appearance of cells exhibiting no reaction to the IFN action, which might mean resistance to IFN. The appearance of resistant cells is explained in [85], [86] by stochastic processes of genetic regulation of interferon response. One cannot exclude that the biphasic pattern of RNA decline under the interferon action is based on the stochastic formation mechanism of cells resistant to the interferon action. This remains an issue for further research.

The role of preexisting mutants in drug-resistant replicon selection is intensively discussed [8], [12]. The central role of preexisting mutations in the acquisition of drug resistance by the HCV replicon was experimentally proved by quantifying the kinetics and uniformity of replication within colonies of drug-resistant fluorescent replicon cells for the NS5B polymerase inhibitor HCV-796 [8]. With the help of our model, we calculated the ratio between the number of mutants in the whole viral RNA pool that are descendants of the preexisting mutants and the number of mutants newly generated in the presence of inhibitor. We also theoretically showed that the preexisting mutants of the NS3 protease play the central role in the acquisition of resistance to the NS3 protease inhibitors by the replicon. As expected, for each mutant type the maximal input of the preexisting mutants into the whole pool of resistant mutant RNAs was observed in the inhibitor concentration range that corresponds to the maximal selective advantage of that mutant.

We used our model to compare the efficacy of the combined action of the NS3 protease inhibitor BILN-2061 with either a potential inhibitor of replicase activity or a potential inhibitor of the host cellular factor production. Our model predicted that the combination of BILN-2061 and the cell factor inhibitor targeting the replicase complex is more effective in reduction of the viral RNA than the combined action of BILN-2061 and a putative host cellular factor production inhibitor.

The calculated kinetics of viral RNA suppression in the presence of both BILN-2061 and a potential inhibitor of the replicase activity are in good agreement with the experimental kinetics of viral RNA suppression under the action of BILN-2061 and the cyclophilin inhibitor NIM811, a derivative of cyclosporine [14]. Cyclophilin is one the key host factors participating in the formation of the active replicase [18]. The derivatives of cyclosporine, which is an inhibitor of the peptidyl-prolylisomerase activity of cyclophilin, are effective in HCV RNA suppression *in vitro* [14], [78], [87], [88] and are currently tested in a clinical trial [89].

We intend to extend our model to predict the efficacy of a new inhibitor alone, or in combination with other inhibitors, or perhaps in multidrug cocktails. The extended model will hopefully unveil the details of the viral RNA synthesis within the vesicles and the mechanism of cellular interferon response, among others. This will bring us closer to design of effective novel antivirals for HCV.

## Supporting Information

Figure S1 **Schematic representation of reactions included into the model.** Designations: *V*, vesicles producing wild type viral RNA; *Vm_11_*, *Vm_22_*, and *Vm_33_*, vesicles producing NS3 protease mutant RNAs; *R*, wild type viral plus-strand RNA; *Rm_11_*, *Rm_22_*, and *Rm_33_*, mutant viral plus-strand RNAs; *polyp*, polyprotein translated from wild type RNA; *polypm_11_*, *polypm_22_*, and *polypm_33_*, polyproteins translated from mutant RNAs *Rm_11_*, *Rm_22_*, and *Rm_33_*, respectively; *p*, viral nonstructural proteins of the wild type; *pm_11_*, *pm_22_*, and *pm_33_*, viral mutant nonstructural proteins; *cf*, cellular factor; *pcf*, replicase formed by the cellular factor *cf* and the wild type viral proteins; *pcfm_11_*, *pcfm_22_*, and *pcfm_33_*, replicase formed by the cellular factor *cf* and mutant viral proteins; *i*, NS3 protease inhibitor; *polypi*, complex of inhibitor with the wild type polyprotein; *polypm_11_i*, *polypm_22_i*, and *polypm_33_i*, complexes of inhibitor with the mutant viral polyproteins. The processes indicated by the arrows have the following rate constants: *kout*, rate constant of the wild type and mutant RNA production by the vesicles *V*, *Vm_11_*, *Vm_22_*, and *Vm_33_*, respectively; *kout_v_vm*, rate constant of the mutant RNA production by the wild type vesicles *V*; *kout_vm_v*, rate constant of the wild type RNA production by the mutant vesicles *Vm_11_*, *Vm_22_*, and *Vm_33_*; *kt*, production rate constant for 1000 viral polyproteins of the wild and mutant types; *kc*, production rate constants for 1000 wild type NS3 proteins; *kcm_11_*, *kcm_22_*, and *kcm_33_*, production rate constants for 1000 mutant NS3 proteins; *kcf*, production rate constant for the cellular factor; *kp*, rate constant of the replicase formation by the cellular factor and by the wild type or mutant viral proteins; *kv*, production rate constant for vesicles *V*, *Vm_11_*, *Vm_22_*, and *Vm_33_*; *ki*, rate constant of interaction between the inhibitor and the wild type polyprotein; *kim_11_, kim_22_*, and *kim_33_*, rate constants of interaction between the inhibitor and the mutant polyproteins, respectively; *ki_obr*, dissociation rate constants for the inhibitor/polyprotein complex for the wild type polyprotein; *ki_obrm_11_*, *ki_obrm_22_*,and *ki_obrm_33_*, dissociation rate constants for the inhibitor/polyprotein complexes for the mutant polyproteins, respectively; *mv*, degradation rate constant for vesicles *V*, *Vm_11_*, *Vm_22_*, and *Vm_33_* (not shown); *mr*, degradation rate constant for the wild type and mutant RNAs (not shown); *mpolyp*, degradation rate constant for the wild type and mutant polyproteins (not shown); *mp*, degradation rate constant for the wild type and mutant nonstructural proteins; *mcf*, degradation rate constant for the cellular factor.
(TIF)

Figure S2 **Viral RNA kinetics in a cell (a) after the subgenomic HCV replicon transfection and (b) after adding 200 nM BILN-2061 inhibitor.** The kinetics was calculated by using deterministic (black line) and stochastic (red line) models.
(TIF)

Figure S3 **Best fitted results for kinetics of the viral RNA decay in the presence of 50 nM BILN-2061 inhibitor in case of the new model with only wild-type HCV RNA (red line), Binder et model** [9] **(blue line) and new model with drug resistant mutant HCV RNAs (black line).**
(TIF)

Table S1 **Propensity functions used in the stochastic model.**
(DOCX)

Text S1 **Model construction.**
(DOC)

Text S2 **Sensitivity to variation of parameter values.**
(PDF)

Text S3 **Method of sensitivity analysis.**
(PDF)

Text S4 **Parameters optimization.**
(PDF)
