# Supplementary figures and images for: A New Stochastic Model for Subgenomic Hepatitis C Virus Replication Considers Drug Resistant Mutants
Source: PLoS One. 2014 Mar 18;9(3):e91502. doi: 10.1371/journal.pone.0091502 (PMC3958367; doi:10.1371/journal.pone.0091502)

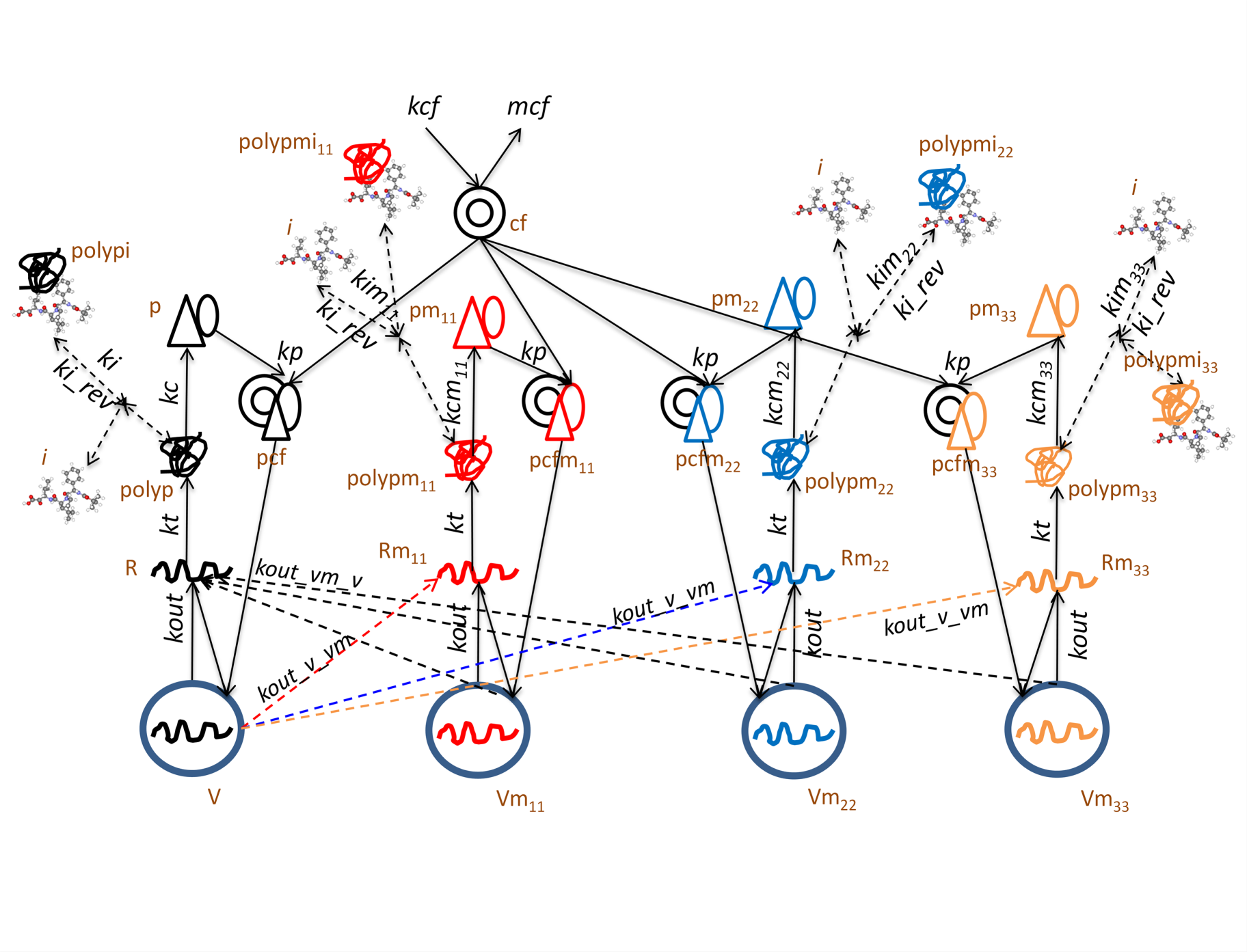

Supplement: Figure S1 — Schematic representation of reactions included into the model. Designations: V, vesicles producing wild type viral RNA; Vm11, Vm22, and Vm33, vesicles producing NS3 protease mutant RNAs; R, wild type viral plus-strand RNA; Rm11, Rm22, and Rm33, mutant viral plus-strand RNAs; polyp, polyprotein translated from wild type RNA; polypm11, polypm22, and polypm33, polyproteins translated from mutant RNAs Rm11, Rm22, and Rm33, respectively; p, viral nonstructural proteins of the wild type; pm11, pm22, and pm33, viral mutant nonstructural proteins; cf, cellular factor; pcf, replicase formed by the cellular factor cf and the wild type viral proteins; pcfm11, pcfm22, and pcfm33, replicase formed by the cellular factor cf and mutant viral proteins; i, NS3 protease inhibitor; polypi, complex of inhibitor with the wild type polyprotein; polypm11i, polypm22i, and polypm33i, complexes of inhibitor with the mutant viral polyproteins. The processes indicated by the arrows have the following rate constants: kout, rate constant of the wild type and mutant RNA production by the vesicles V, Vm11, Vm22, and Vm33, respectively; kout_v_vm, rate constant of the mutant RNA production by the wild type vesicles V; kout_vm_v, rate constant of the wild type RNA production by the mutant vesicles Vm11, Vm22, and Vm33; kt, production rate constant for 1000 viral polyproteins of the wild and mutant types; kc, production rate constants for 1000 wild type NS3 proteins; kcm11, kcm22, and kcm33, production rate constants for 1000 mutant NS3 proteins; kcf, production rate constant for the cellular factor; kp, rate constant of the replicase formation by the cellular factor and by the wild type or mutant viral proteins; kv, production rate constant for vesicles V, Vm11, Vm22, and Vm33; ki, rate constant of interaction between the inhibitor and the wild type polyprotein; kim11, kim22, and kim33, rate constants of interaction between the inhibitor and the mutant polyproteins, respectively; ki_obr, dissociatio [file pone.0091502.s001.tif]

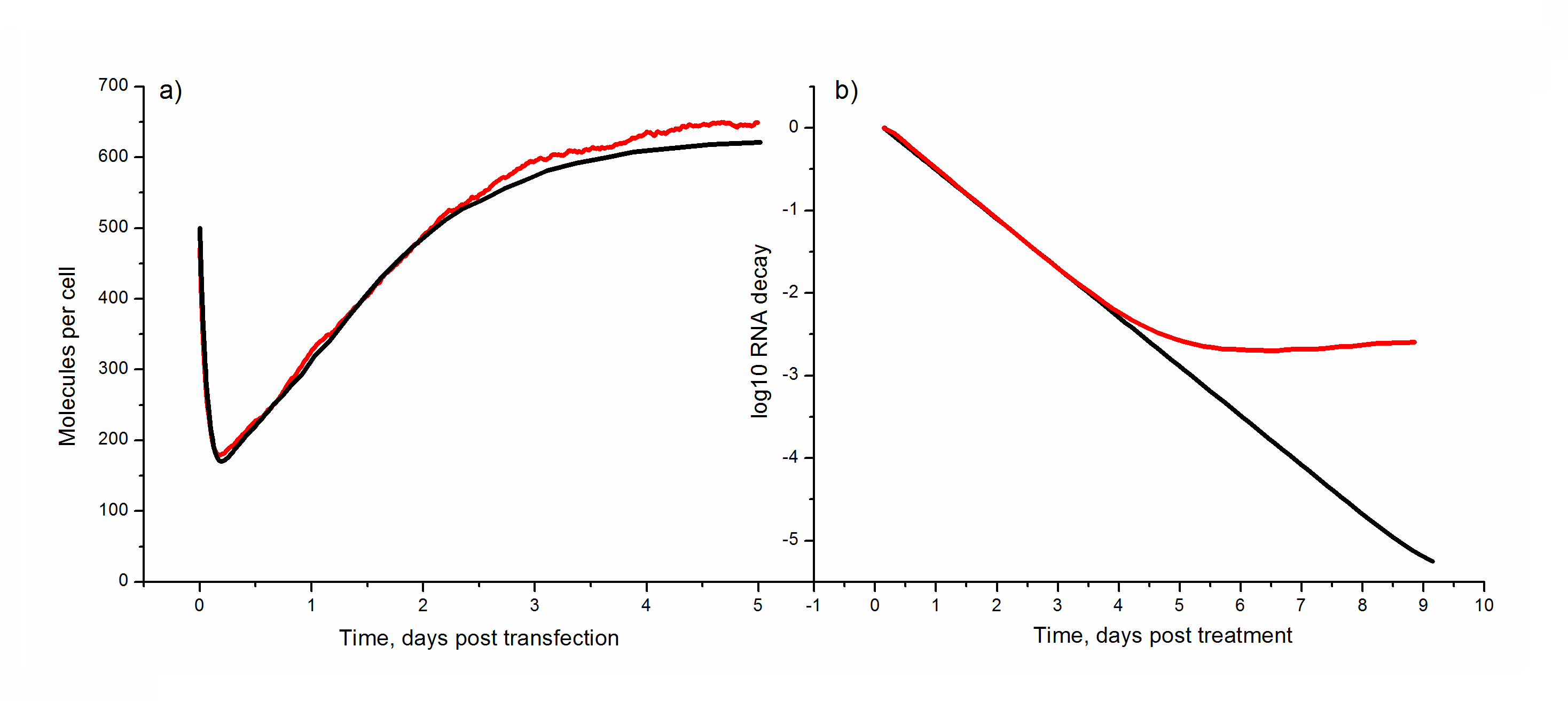

Supplement: Figure S2 — Viral RNA kinetics in a cell (a) after the subgenomic HCV replicon transfection and (b) after adding 200 nM BILN-2061 inhibitor. The kinetics was calculated by using deterministic (black line) and stochastic (red line) models. (TIF) [file pone.0091502.s002.tif]
