# Supplementary material for: A New Stochastic Model for Subgenomic Hepatitis C Virus Replication Considers Drug Resistant Mutants
Source: PLoS One. 2014 Mar 18;9(3):e91502. doi: 10.1371/journal.pone.0091502 (PMC3958367; doi:10.1371/journal.pone.0091502)
Supplement: Table S1 — Propensity functions used in the stochastic model. (DOCX) [file pone.0091502.s004.docx]

**Table S1. Propensity functions used in the stochastic model**

|  | | | | | |
| --- | --- | --- | --- | --- | --- |
| kv·R·pcf | V→V+1 pcf→pcf-1 | Formation of vesicles with wild type viral RNA | kv·Rm1·pcfm1 | pcfm1→pcfm1-1 Rm1→Rm1-1 Vm1→Vm1+1 | Formation of vesicles containing mutant viral RNA from group 1 |
| mv·V | V→V-1 | Degradation of vesicles with wild type viral RNA | mv·Vm1 | Vm1→Vm1-1 | Degradation of vesicles containing mutant 1 viral RNA form |
| kout·V | R→R+1 | Production of wild type RNA | kout·Vm1 | Rm1→Rm1+1 | Production of mutant 1 RNA by vesicles containing mutant 1 viral RNA form |
| mr·R | R→R-1 | Degradation of wild type RNA | mr·Rm1 | Rm1→Rm1-1 | Degradation of mutant 1 viral RNA |
| kc·polyp | polyp→polyp-1 p→p+1 | Processing of wild type polyprotein | kcm1·polypm1 | polypm1->polypm1-1 pm1→pm1+1 | Processing of mutant 1 polyprotein |
| kp·p·cf | pcf→pcf+1 p→p-1 cf→cf-1 | Formation of replicase containing viral nonstructural proteins translated from wild type viral RNA | kp·pm1·cf | pcfm1→pcfm1+1 pm1→pm1+1 cf→cf-1 | Formation of replicase containing viral nonstructural proteins translated from mutant 1 viral RNA form |
| mp·p | p→p-1 | Degradation of nonstructural proteins translated from wild type viral RNA | mp·pm1 | pm1→pm1-1 | Degradation of nonstructural proteins translated from mutant 1 viral RNA form |
| mv·pcf | pcf→pcf-1 | Degradation of replicase containing viral nonstructural proteins translated from wild type viral RNA | mv·pcfm1 | pcfm1→pcfm1-1 | Degradation of replicase containing viral nonstructural proteins translated from mutant 1 RNA |
| kt·R | polyp→polyp+1 | Translation of wild type polyprotein | kt·Rm1 | polypm1→polypm1+1 | Translation of mutant 1 polyprotein |
| mpolyp·polyp | polyp→polyp-1 | Degradation of wild type polyprotein | mpolyp·polypm1 | polypm1→polypm1-1 | Degradation of mutant 1 polyprotein |
| kout_m·V | Rm1→Rm1+1 | Production of mutant 1 RNA by vesicles containing wild type viral RNA form | kout_m·Vm1 | R→R+1 | Production of wt RNA by vesicles containing mutant 1 viral RNA form |
| mcf·cf | cf→cf-1 | Cellular factor degradation | kcf | cf→cf+1 | Production of cellular factor |
| ki·polyp·i | polyp→polyp-1 polypi→polypi+1 | Non-covalent polyprotein (wild type)/inhibitor complex formation | kim1·polypm1·i | polypm1→polypm1-1 polypmi1→polypmi1+1 | Non-covalent polyprotein (mutant 1)/inhibitor complex formation |
| kirev·polypi | polyp→polyp+1 polypi→polypi-1 | Non-covalent polyprotein (wild type)/inhibitor complex dissociation | kirev·polypmi1 | polypm1→ polypm1+1 polypmi1→ polypmi1-1 | Non-covalent polyprotein (mutant 1)/inhibitor complex dissociation |
| mpolyp·polypi | polypi→polypi-1 | Non-covalent polyprotein (wild type)/inhibitor complex degradation | mpolyp·polypmi1 | polypmi1→ polypmi1-1 | Non-covalent polyprotein (mutant 1)/inhibitor complex degradation |
| ki2·polypi | polypi→polypi-1 polypi2→polypi2+1 | Covalent polyprotein (wild type)/inhibitor complex formation | ki2·polypmi1 | polypmi1→ polypmi1-1 polypmi21→ polypmi21+1 | Covalent polyprotein (mutant 1) -inhibitor complex formation |
| kirev2·polypi2 | polypi→polypi+1 polypi2→polypi2-1 | Dissociation of the covalent polyprotein (wild type) -inhibitor complex | kirev2·polypmi21 | polypmi1→ polypmi1+1 polypmi21→ polypmi21-1 | Dissociation to the non-covalent polyprotein (mutant 1)/inhibitor complex |
| mpolyp·polypi2 | polypi2→polypi2-1 | Covalent polyprotein (wild type)-inhibitor complex degradation | mpolyp·polypmi21 | polypmi21→ polypmi21-1 | Covalent polyprotein (mutant 1)/inhibitor complex degradation |
