# Supplementary material for: A New Stochastic Model for Subgenomic Hepatitis C Virus Replication Considers Drug Resistant Mutants
Source: PLoS One. 2014 Mar 18;9(3):e91502. doi: 10.1371/journal.pone.0091502 (PMC3958367; doi:10.1371/journal.pone.0091502)
Supplement: Text S1 — Model construction. (DOC) [file pone.0091502.s005.doc]

**Text S1. Model construction**

**Nikita V. Ivanisenko et al.**

Schematic representation of reactions included into the model (Fig.S1).

**Fig. S1.** Schematic representation of reactions included into the model. Designations: *V,* vesicles producing wild type viral RNA; *Vm_11_,* *Vm_22_,* and *Vm_33_,* vesicles producing NS3 protease mutant RNAs; *R*, wild type viral plus-strand RNA; *Rm_11_,* *Rm_22_,* and *Rm_33_*, mutant viral plus-strand RNAs; *polyp,* polyprotein translated from wild type RNA; *polypm_11_,* *polypm_22_,* and *polypm_33_,* polyproteins translated from mutant RNAs *Rm_11_,* *Rm_22_,* and *Rm_33_,* respectively; *p,* viral nonstructural proteins of the wild type; *pm_11_,* *pm_22_,* and *pm_33_,* viral mutant nonstructural proteins; *сf*, cellular factor; *pcf,* replicase formed by the cellular factor *сf* and the wild type viral proteins; *pcfm_11_,* *pcfm_22_,* and *pcfm_33_*, replicase formed by the cellular factor *сf* and mutant viral proteins; *i*, NS3 protease inhibitor; *polypi*, complex of inhibitor with the wild type polyprotein; *polypm_11_i,* *polypm_22_i,* and *polypm_33_i,* complexes of inhibitor with the mutant viral polyproteins. The processes indicated by the arrows have the following rate constants: *kout*, rate constant of the wild type and mutant RNA production by the vesicles *V,* *Vm_11_,* *Vm_22_,* and *Vm_33_*, respectively; *kout_v_vm,* rate constant of the mutant RNA production by the wild type vesicles *V*; *kout_vm_v,* rate constant of the wild type RNA production by the mutant vesicles *Vm_11_,* *Vm_22_,* and *Vm_33_*; *kt,* production rate constant for 1000 viral polyproteins of the wild and mutant types; *kc*, production rate constants for 1000 wild type NS3 proteins; *kcm_11_*, *kcm_22_*, and *kcm_33_,* production rate constants for 1000 mutant NS3 proteins; *kcf*, production rate constant for the cellular factor; *kp*, rate constant of the replicase formation by the cellular factor and by the wild type or mutant viral proteins; *kv*, production rate constant for vesicles *V,* *Vm_11_,* *Vm_22_,* and *Vm_33_*; *ki*, rate constant of interaction between the inhibitor and the wild type polyprotein; *kim_11_, kim_22_,* and *kim_33_,* rate constants of interaction between the inhibitor and the mutant polyproteins , respectively; *ki_obr*, dissociation rate constants for the inhibitor/polyprotein complex for the wild type polyprotein; *ki_obrm_11_, ki_obrm_22_,*and *ki_obrm_33_*, dissociation rate constants for the inhibitor/polyprotein complexes for the mutant polyproteins, respectively; *mv*, degradation rate constant for vesicles *V,* *Vm_11_,* *Vm_22_,* and *Vm_33_* (not shown); *mr*, degradation rate constant for the wild type and mutant RNAs (not shown); *mpolyp*, degradation rate constant for the wild type and mutant polyproteins (not shown); *mp*, degradation rate constant for the wild type and mutant nonstructural proteins; *mcf*, degradation rate constant for the cellular factor.

**Table S1. Propensity functions used in the stochastic** model

|  | | | | | |
| --- | --- | --- | --- | --- | --- |
| kv·R·pcf | V→V+1 pcf→pcf-1 | Formation of vesicles with wild type viral RNA | kv·Rm1·pcfm1 | pcfm1→pcfm1-1  Rm1→Rm1-1 Vm1→Vm1+1 | Formation of vesicles containing mutant viral RNA from group 1 |
| mv·V | V→V-1 | Degradation of vesicles with wild type viral RNA | mv·Vm1 | Vm1→Vm1-1 | Degradation of vesicles containing mutant 1 viral RNA form |
| kout·V | R→R+1 | Production of wild type RNA | kout·Vm1 | Rm1→Rm1+1 | Production of mutant 1 RNA by vesicles containing mutant 1 viral RNA form |
| mr·R | R→R-1 | Degradation of wild type RNA | mr·Rm1 | Rm1→Rm1-1 | Degradation of mutant 1 viral RNA |
| kc·polyp | polyp→polyp-1  p→p+1 | Processing of wild type polyprotein | kcm1·polypm1 | polypm1->polypm1-1 pm1→pm1+1 | Processing of mutant 1 polyprotein |
| kp·p·cf | pcf→pcf+1 p→p-1  cf→cf-1 | Formation of replicase containing viral nonstructural proteins translated from wild type viral RNA | kp·pm1·cf | pcfm1→pcfm1+1 pm1→pm1+1 cf→cf-1 | Formation of replicase containing viral nonstructural proteins translated from mutant 1 viral RNA form |
| mp·p | p→p-1 | Degradation of nonstructural proteins translated from wild type viral RNA | mp·pm1 | pm1→pm1-1 | Degradation of nonstructural proteins translated from mutant 1 viral RNA form |
| mv·pcf | pcf→pcf-1 | Degradation of replicase containing viral nonstructural proteins translated from wild type viral RNA | mv·pcfm1 | pcfm1→pcfm1-1 | Degradation of replicase containing viral nonstructural proteins translated from mutant 1 RNA |
| kt·R | polyp→polyp+1 | Translation of wild type polyprotein | kt·Rm1 | polypm1→polypm1+1 | Translation of mutant 1 polyprotein |
| mpolyp·polyp | polyp→polyp-1 | Degradation of wild type polyprotein | mpolyp·polypm1 | polypm1→polypm1-1 | Degradation of mutant 1 polyprotein |
| kout_m·V | Rm1→Rm1+1 | Production of mutant 1 RNA by vesicles containing wild type viral RNA form | kout_m·Vm1 | R→R+1 | Production of wt RNA by vesicles containing mutant 1 viral RNA form |
| mcf·cf | cf→cf-1 | Cellular factor degradation | kcf | cf→cf+1 | Production of cellular factor |
| ki·polyp·i | polyp→polyp-1 polypi→polypi+1 | Non-covalent polyprotein (wild type)/inhibitor complex formation | kim1·polypm1·i | polypm1→polypm1-1 polypmi1→polypmi1+1 | Non-covalent polyprotein (mutant 1)/inhibitor complex formation |
| kirev·polypi | polyp→polyp+1 polypi→polypi-1 | Non-covalent polyprotein (wild type)/inhibitor complex dissociation | kirev·polypmi1 | polypm1→ polypm1+1 polypmi1→ polypmi1-1 | Non-covalent polyprotein (mutant 1)/inhibitor complex dissociation |
| mpolyp·polypi | polypi→polypi-1 | Non-covalent polyprotein (wild type)/inhibitor complex degradation | mpolyp·polypmi1 | polypmi1→ polypmi1-1 | Non-covalent polyprotein (mutant 1)/inhibitor complex degradation |
| ki2·polypi | polypi→polypi-1 polypi2→polypi2+1 | Covalent polyprotein (wild type)/inhibitor complex formation | ki2·polypmi1 | polypmi1→ polypmi1-1 polypmi21→ polypmi21+1 | Covalent polyprotein (mutant 1) -inhibitor complex formation |
| kirev2·polypi2 | polypi→polypi+1 polypi2→polypi2-1 | Dissociation of the covalent polyprotein (wild type) -inhibitor complex | kirev2·polypmi21 | polypmi1→ polypmi1+1 polypmi21→ polypmi21-1 | Dissociation to the non-covalent polyprotein (mutant 1)/inhibitor complex |
| mpolyp·polypi2 | polypi2→polypi2-1 | Covalent polyprotein (wild type)-inhibitor complex degradation | mpolyp·polypmi21 | polypmi21→ polypmi21-1 | Covalent polyprotein (mutant 1)/inhibitor complex degradation |

**ODEs used in deterministic model:**

| $\frac{dV}{dt}=kv\cdot R\cdot pcf-mv\cdot V$ | (1) |
| --- | --- |
| $\frac{dR}{dt}=kout\cdot V+\sum_{i} kout\_m\cdot Vm_{i}-kv\cdot R\cdot pcf-mr\cdot R$ | (2) |
| $\frac{dp}{dt}=kc\cdot polyp-kp\cdot p\cdot cf-mp\cdot p$ | (3) |
| $\frac{dpcf}{dt}=kp\cdot p\cdot cf-kv\cdot R\cdot pcf-mv\cdot pcf$ | (4) |
| $\frac{dVm_{i}}{dt}=kv\cdot Rm_{i}\cdot pcfm_{i}-mv\cdot Vm_{i}$ | (5) |
| $\frac{dRm_{i}}{dt}=kout\cdot Vm_{i}+kout\_m\cdot V-kv\cdot Rm_{i}\cdot pcfm_{i}-mr\cdot Rm_{i}$ | (6) |
| $\frac{dpm_{i}}{dt}=kcm\cdot polypm_{i}-kp\cdot pm_{i}\cdot cf-mp\cdot pm_{i}$ | (7) |
| $\frac{dpcfm_{i}}{dt}=kp\cdot pm_{i}\cdot cf-kv\cdot Rm_{i}\cdot pcfm_{i}-mv\cdot pcfm_{i}$ | (8) |
| $\frac{dcf}{dt}=kcf-kp\cdot(p+pm_{i})\cdot cf-mcf\cdot cf$ | (9) |
| $\frac{dpolyp}{dt}=kt\cdot R-kc\cdot polyp-ki\cdot polyp+ki\_rev\cdot polypi-mpolyp\cdot polyp$ | (10) |
| $\frac{dpolypi}{dt}=ki\cdot polyp-ki\_rev\cdot polypi-ki2\cdot polypi+ki\_rev2\cdot polypi*-mpolyp\cdot polypi$ | (11) |
| $\frac{dpolypi*}{dt}=ki2\cdot polypi-ki\_rev2\cdot polypi*-mpolyp\cdot polypi*$ | (12) |
| $\frac{dpolypm_{i}}{dt}=kt\cdot R-kc\cdot polypm_{i}-ki\cdot polypm_{i}+ki\_rev\cdot polypmi_{i}-mpolyp\cdot polypm_{i}$ | (13) |
| $\frac{dpolypmi_{i}}{dt}=ki\cdot polypm_{i}-ki\_rev\cdot polypmi_{i}-ki2\cdot polypmi_{i}+ki\_rev2\cdot polypmi_{i}*-mpolyp\cdot polypmi_{i}$ | (14) |
| $\frac{dpolypmi_{i}*}{dt}=ki2\cdot polypmi_{i}-ki\_rev2\cdot polypmi_{i}*-mpolyp\cdot polypmi_{i}*$ | (15) |

**Fig. S2** Viral RNA kinetics in a cell (a) after the subgenomic HCV replicon transfection and (b) after adding 200 nM BILN-2061 inhibitor. The kinetics was calculated by using deterministic (black line) and stochastic (red line) models.

We have performed several fits using special version of our new model that includes only wild type HCV mRNA but without resistant mutants, trying to obtain parameters that describe the data for 3, 6, and 9 days after treatment with inhibitors. Note that the second phase of mRNA decay is already observed in the experiment on the 6 and 9 days.

**Fig. S3** Best fitted results for kinetics of the viral RNA decay in the presence of 50 nM BILN-2061 inhibitor in case of the new model with only wild-type HCV RNA (red line), Binder et model [9] (blue line) and new model with drug resistant mutant HCV RNAs (black line).

The resulting fits had poor quality (see Fig. S3).

To compare the models quantitatively, we used the Akaike information criterion,

AIC = *n* ln(RSS/*n*) + 2*k*,

where *n* is the number of data points, *k* is the number of parameters, and RSS is the residual sum of squares, the value of objective function. For the model without resistant mutants RSS = 10.592758 and AIC = 27*ln(10.592758/27)+2*11 = -3.26299, and for the model with resistant mutants RSS = 4.83679 and AIC = 27*ln(4.83679/27)+2*22 = -2.428811.

The corresponding values in the case of *k* being equal to the number of parameters obtained by fitting only are as follows: AIC = 27*ln(10.592758/27)+2*7 = -11.26299, and for the model with resistant mutants AIC = 27*ln(4.83679/27)+2*18 = -10.42881.

Therefore, the value of RSS for the model with only wild-type HCV RNA is more than 2 times bigger than the RSS value for the model that considers resistant mutants. Furthermore, based on the comparison of the AIC values we conclude that the model that includes resistant mutants is more adequate for description of the observed experimental data (Fig. S3).

Additionally, Fig. S3 shows that the Binder et al. model [9], which does not include drug-resistant mutants, also has no advantage in the description of kinetics for RNA suppression. For these simulations the model was modified by introduction of NS3 protease inhibitors (data not shown).

**References**

1. Pietschmann T., Lohmann V., Rutter G., Kurpanek K., Bartenschlager R. Characterization of cell lines carrying self-replicating hepatitis C virus RNAs. *J Virol.* 2001. V. 75. P. 1252-1264.
2. Li Y., Masaki T., Yamane D., McGivern D.R., Lemon S.M. Competing and noncompeting activities of miR-122 and the 5' exonuclease Xrn1 in regulation of hepatitis C virus replication. *Proc Natl Acad Sci USA*. 2013. V. 110. P. 1881-1886
3. Dahari H., Ribeiro R.M., Rice C.M., Perelson A.S. Mathematical modeling of subgenomic hepatitis C viral replication in Huh-7 cells. *J Virol.* 2007. V. 81. P. 750–760.
4. Lin C., Pragai B.M., Grakoui A., Xu J., Rice C.M. Hepatitis C virus NS3 serine proteinase: trans-cleavage requirements and processing kinetics. *J Virol*. 1994. V. 68. P. 8147-1857.
5. Powdrill M.H., Tchesnokov E.P., Kozak R.A., Russell R.S., Martin R., Svarovskaia E.S., Mo H., Kouyos R.D., Götte M. Contribution of a mutational bias in hepatitis C virus replication to the genetic barrier in the development of drug resistance. [*Proc Natl Acad* *Sci USA*.](http://www.ncbi.nlm.nih.gov/pubmed?term=proc natl acad sci usa 2011 20509-20513) 2011. V. 108. P. 20509-20513.
6. Flores M.V., Strawbridge J., Ciaramella G., Corbau R. HCV-NS3 inhibitors: determination of their kinetic parameters and mechanism. *Biochim Biophys Acta*. 2009. V. 1794. P. 1441-1448.
7. He Y., King M.S., Kempf D.J., Lu L., Lim H.B., Krishnan P., Kati W., Middleton T., Molla A. Relative replication capacity and selective advantage profiles of protease inhibitor-resistant hepatitis C virus (HCV) NS3 protease mutants in the HCV genotype 1b replicon system. *Antimicrob Agents Chemother.* 2008. V. 52. P. 1101-1110.
8. Tong X., Chase R., Skelton A., Chen T., Wright-Minogue J., Malcolm B.A. Identification and analysis of fitness of resistance mutations against the HCV protease inhibitor SCH 503034. *Antiviral Res*. 2006. V. 70. P. 28-38.
9. M. Binder, N. Sulaimanov, D. Clausznitzer, M. Schulze, C. M. Hueber, S. Lenz, J. Schloeder, M. Trippler, R. Bartenschlager, V. Lohmann, L. Kaderali. Replication Vesicles are Load- and Choke-Points in the Hepatitis C Virus Lifecycle, *PLoS Pathogens*, 2013. V. 9. P. e1003561.
