# Supplementary material for: A New Stochastic Model for Subgenomic Hepatitis C Virus Replication Considers Drug Resistant Mutants
Source: PLoS One. 2014 Mar 18;9(3):e91502. doi: 10.1371/journal.pone.0091502 (PMC3958367; doi:10.1371/journal.pone.0091502)
Supplement: Text S2 — Sensitivity to variation of parameter values. (PDF) [file pone.0091502.s006.pdf]

Text S2. Sensitivity to variation of parameter values

1. Variation of each single parameter

We calculated values of the objective functional including all penalizing terms (Cost) when each parameter was varied around its optimal value with all the rest parameters being fixed at the optimal values. The figures below show variation of the full cost and its components for each single parameter varied in the limits from 0.5 to 1.5 of the parameter optimal value, for 6 sets of optimal parameter values found by optimization.

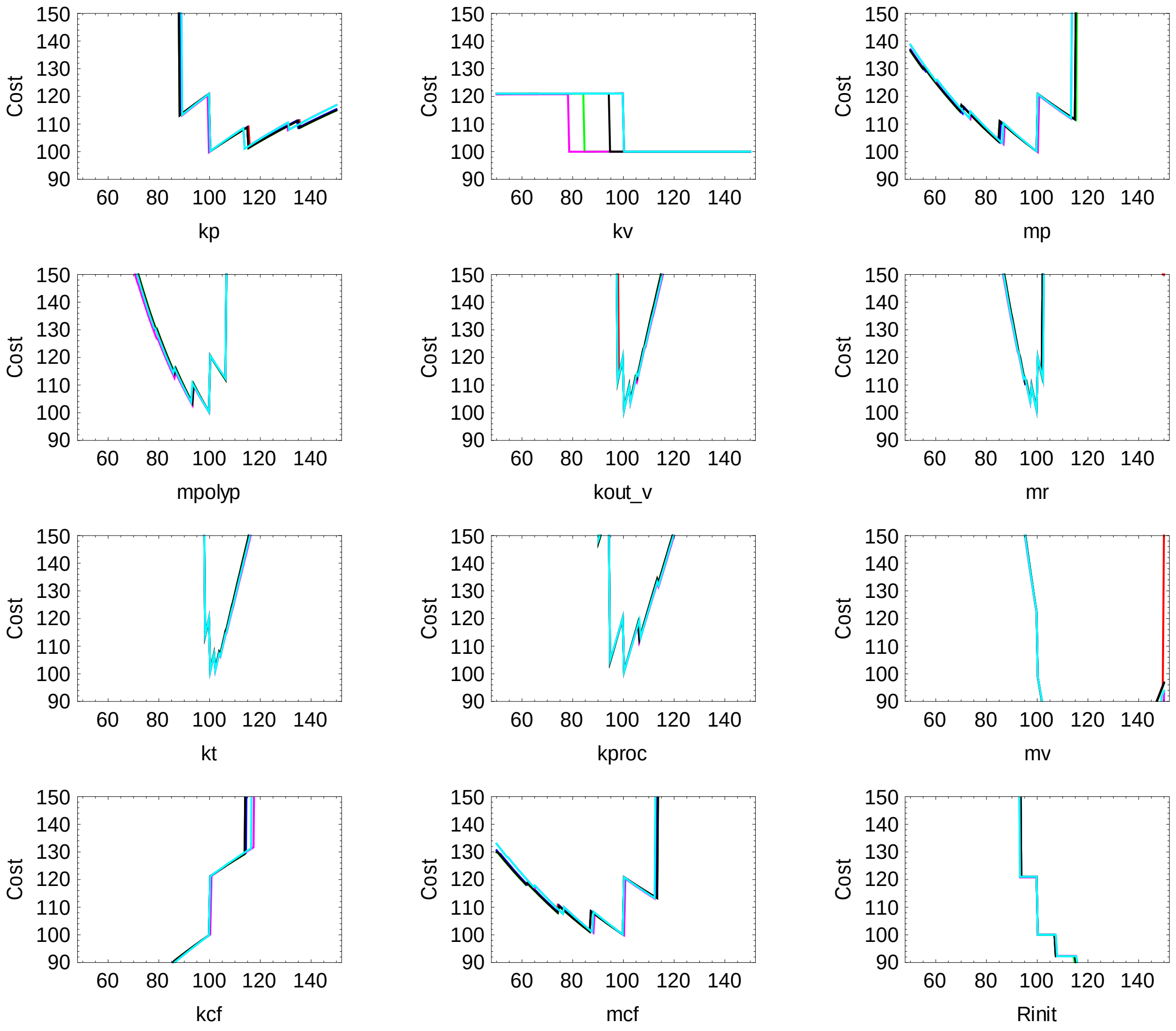

Fig. S3. The full cost as a function of each parameter with all other parameters fixed at the optimal values. Different colors correspond to 6 different sets of optimal parameter values. The cost and parameter values are given in percent of their optimal values (i.e., 100% in the figure correspond to the optimal values).

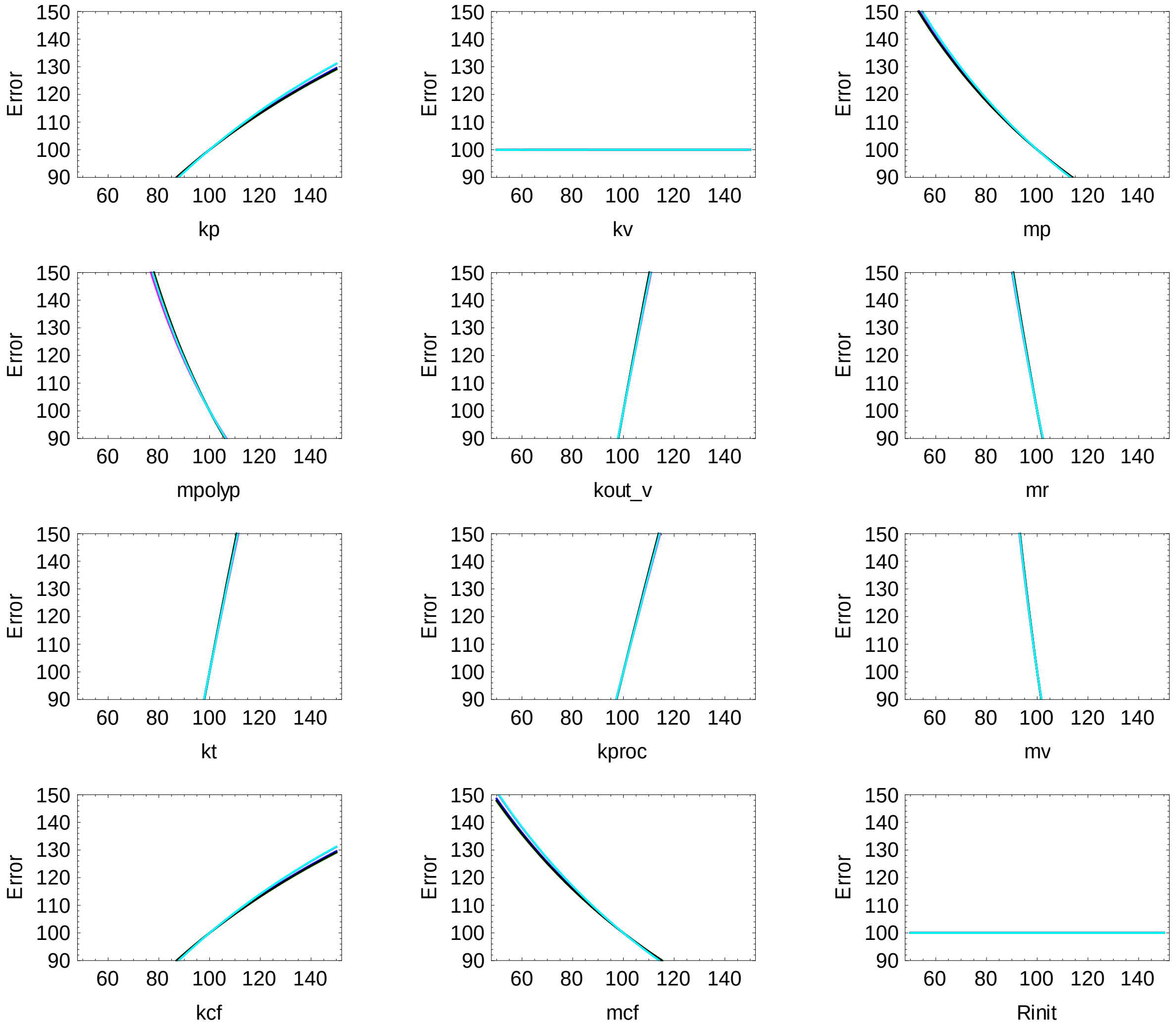

Fig. S4. The same as in Fig. S3 but for the Error part of the full cost, representing the root-mean-squared deviation between solution and data.

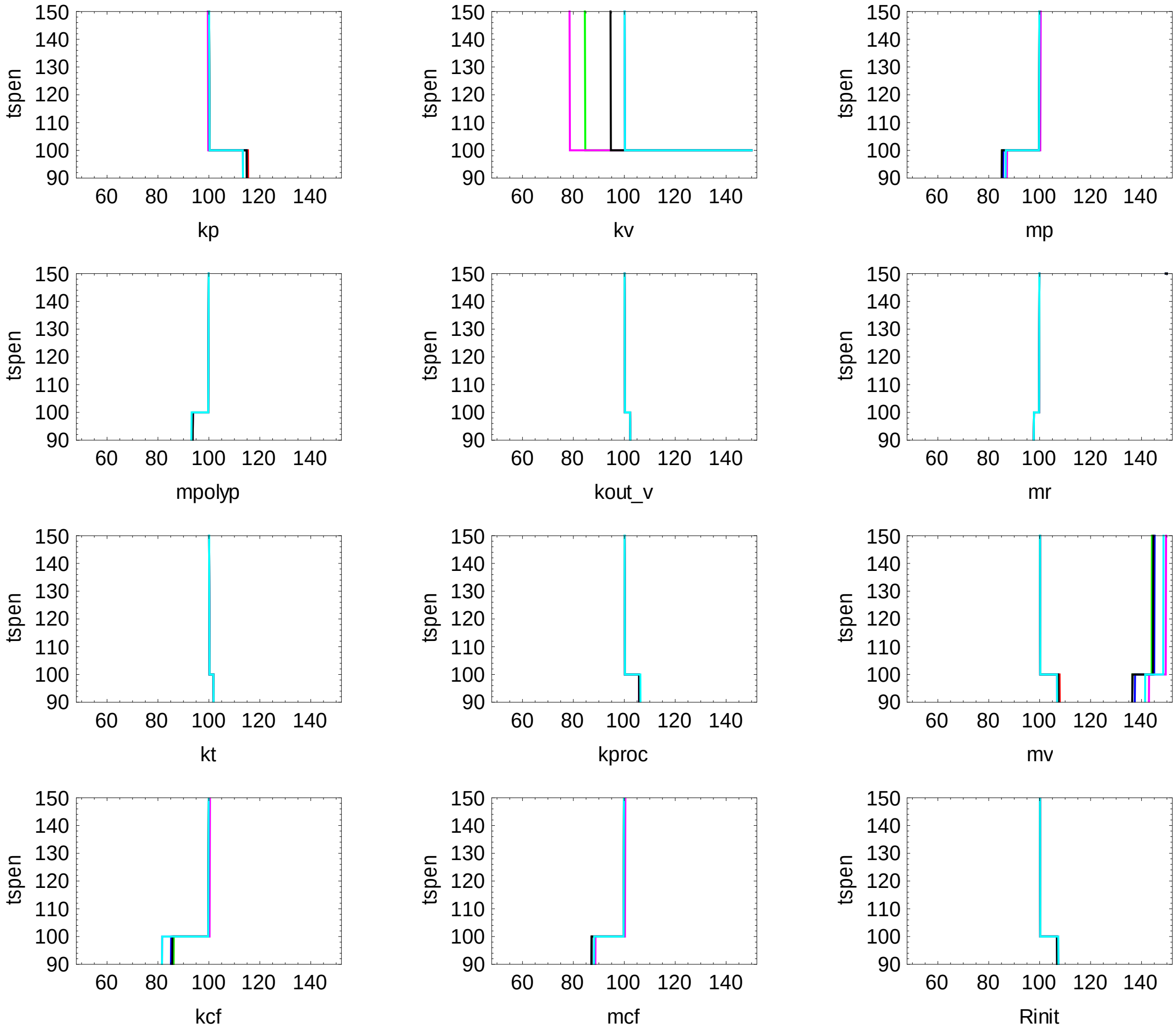

Fig. S5. The same as in Fig. S3 but for the tspen part of the full cost, representing a penalty for the time to reach the stationary level.

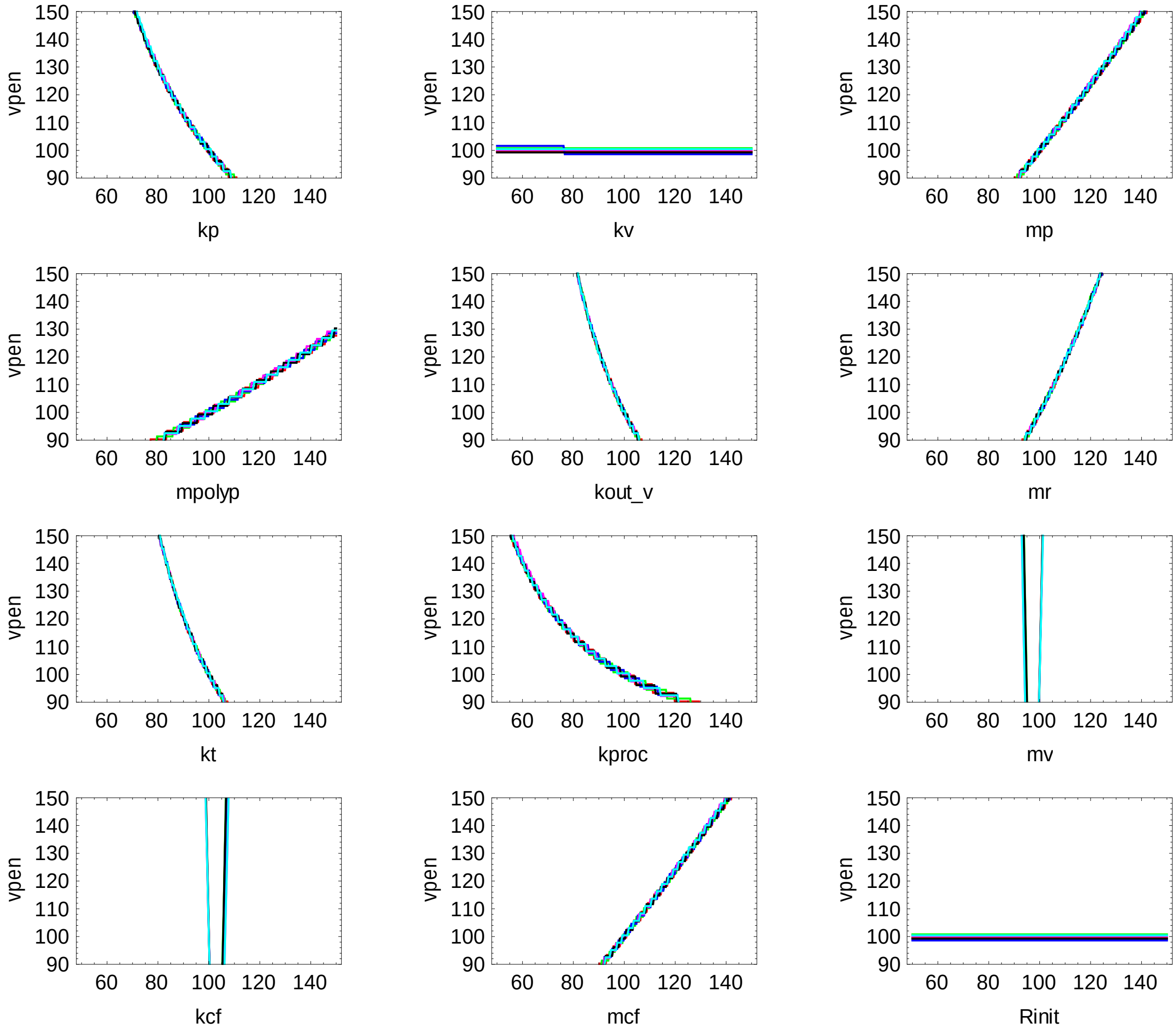

Fig. S6. The same as in Fig. S3 but for the vpen part of the full cost, representing a penalty for the number of vesicles.

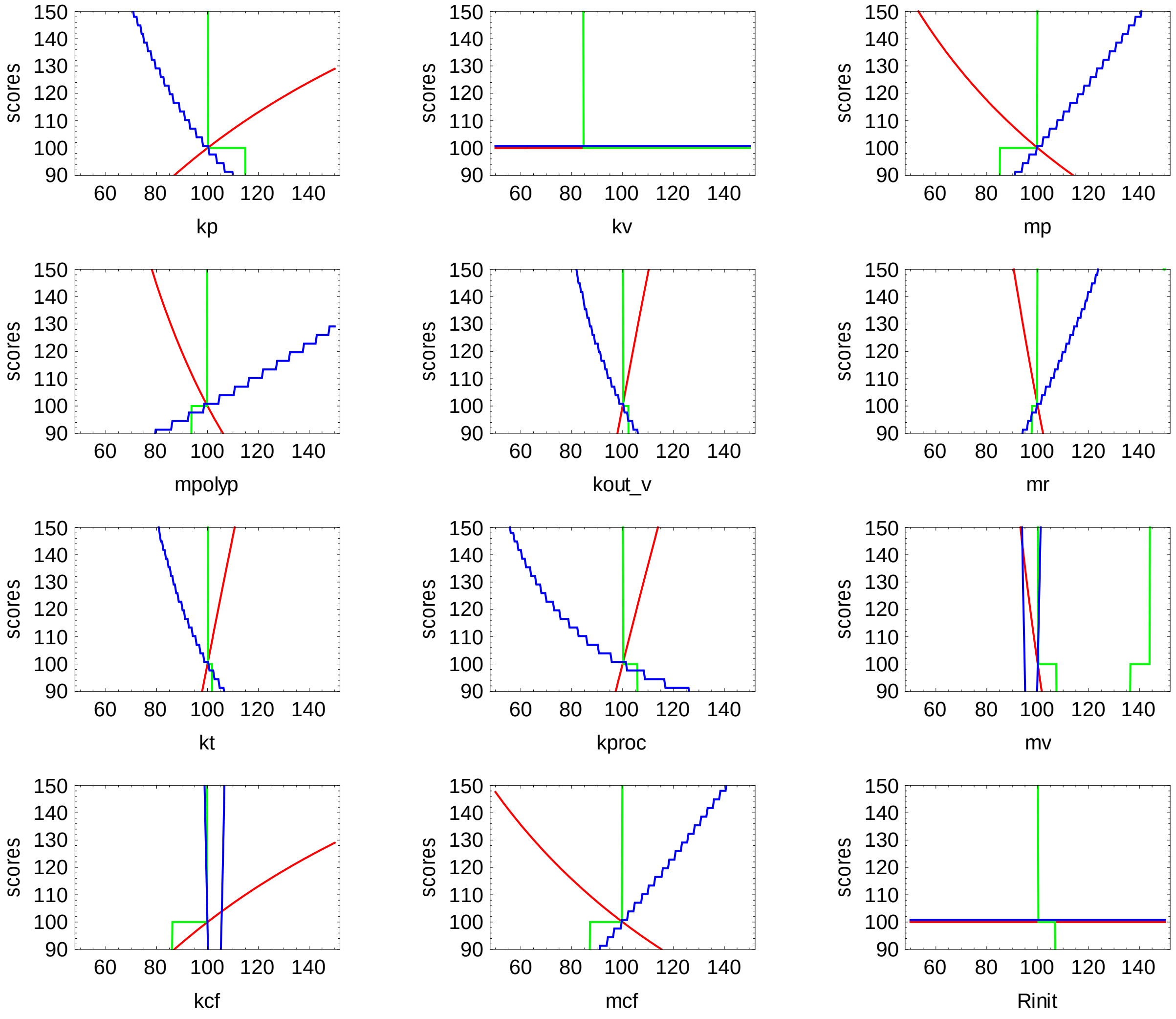

Fig. S7. The three scores simultaneously (red for error, green for tspen, and blue for vpen) for one set of the optimal parameter values. The axes show the scores and parameter values in percent of their optimal values.

2. Box calculations

By applying a method described in Supplementary Text 3, we calculated a vicinity of the optimal parameter values in the form of a multi-dimensional box in the parameter space such that about 80% of its volume correspond to model solutions whose cost deviates from the optimal cost value less than a predefined quantity. We performed the calculations for this quantity equal to 10%, 20%, 30%, 40%, and 50% of the optimal cost value. The figure below shows results for three sets of the optimal parameter values found by optimization.

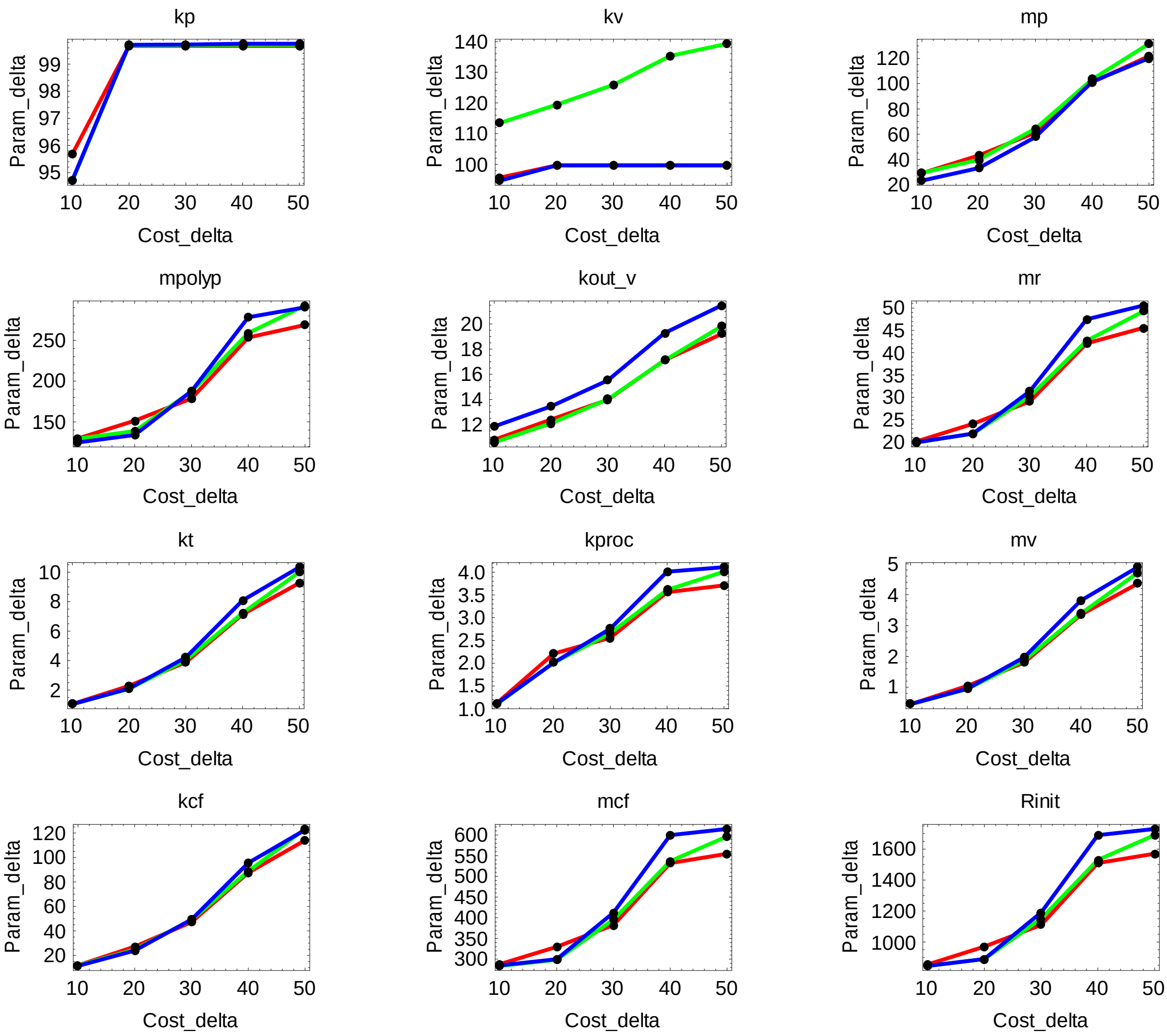

Fig. S8. Characteristics of the multi-dimensional box around the optimal parameter values. Each panel represents allowed ranges (Param_delta) inside the box for each parameter in percent of the optimal value for that parameter. The box is defined as the one having about 80% (more precisely, from 79% to 85%) of its volume corresponding to the model solutions whose cost is inside the prescribed variation limits given by Cost_delta. For example, Cost_delta = 10% means that the cost of the solutions in the box is allowed to deviate from the optimal cost value not more than by 10% of that value. The cost is the logarithm of the squared deviation of the model solution from the data penalized by scaled logarithm of difference between the stationary number of vesicles in the model and the optimal number of vesicles equal to 100. Different colors correspond to three different sets of optimal parameter values.

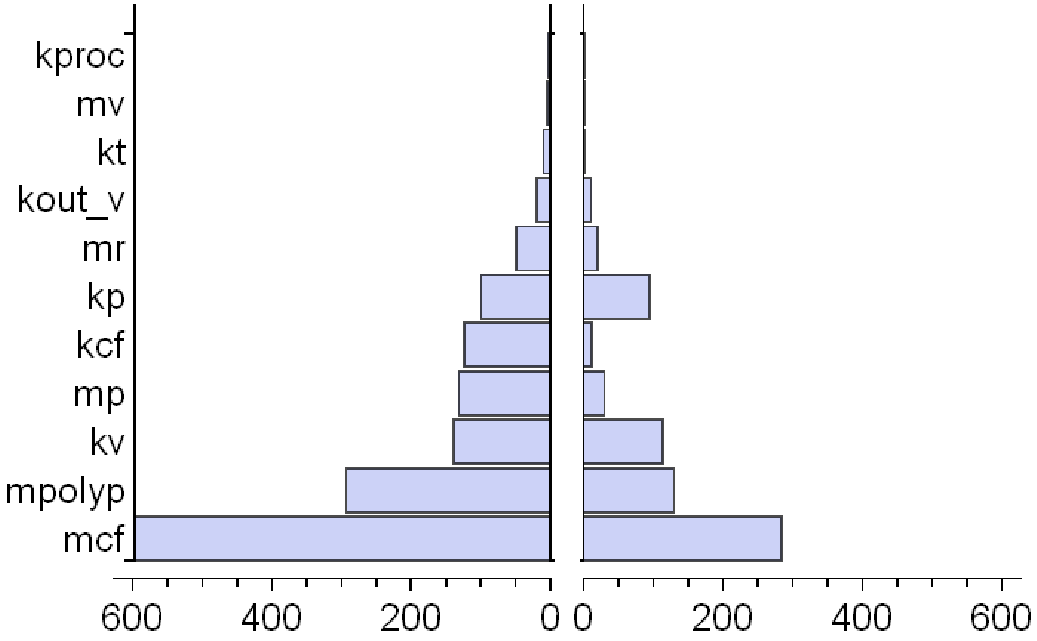

Fig. S9 The bar chart of the allowed parameter variation (in percent of the optimal parameter values) following from the calculated multi-dimensional box from Fig. S8, for one set of the optimal parameter values. Left part is for 50% variation allowed for the cost (Cost_delta = 50 in Fig. S8), and right for 10%. The ordering of the parameters in the figure is in decreasing value for the 50%-case. The parameter describing the initial value for the solution (Rinit) was excluded in the graph for better visibility of the numbers for the rest parameters.
