# Supplementary material for: A New Stochastic Model for Subgenomic Hepatitis C Virus Replication Considers Drug Resistant Mutants
Source: PLoS One. 2014 Mar 18;9(3):e91502. doi: 10.1371/journal.pone.0091502 (PMC3958367; doi:10.1371/journal.pone.0091502)
Supplement: Text S3 — Method of sensitivity analysis. (PDF) [file pone.0091502.s007.pdf]

1 Methods

Here we will describe how one can find a domain of "good" initial data and parameters around a point that is *a priori* known to be "good".

1.1 The goal

Consider an autonomous system of differential equations on $\mathbb{R}^n$ depending on initial data $u_0 \in \mathbb{R}^n$ and parameters $\theta \in \mathbb{R}^{m_0}$

$$\dot{u}(t, u_0, \theta) = f(u(t, u_0, \theta)) \tag{1}$$

where f is good enough (e.g. f is smooth).

We are interested in varying some components of initial data vector and some parameters. We will call them variables. Assume that we fixed all non-variables. Than we consider a solution as a curve $\alpha(\lambda)$ in $\mathbb{R}^n$ that depends only on a vector $\lambda \in \mathbb{R}^m$ of variables.

Let F be a functional mapping a smooth curve in $\mathbb{R}^n$ to a nonnegative number, i.e.

$$F : C^1(\mathbb{R} \to \mathbb{R}^n) \to [0, +\infty).$$

We think of F as a measure of defectiveness – the bigger it is, the worse situation we have. We assume that F is continuous and differentiable (in Fréchetsense).

Denote $g(x) = F(\alpha(x))$.

Let Q be a positive number. We will call a vector λ good if $g(\lambda) \leq Q$.

Assume that we know that λ_0 is good. We want to find a box-shaped neighborhood V of λ_0 in $\mathbb{R}^m$ that contains mostly good vectors. "Mostly" here means that we need the ratio between $\mathrm{Vol}\,(\{v \in V \mid v \text{ is good}\})$ and $\mathrm{Vol}\,(V)$ to be close to 1, i.e. greater than some predefined $\varkappa < 1$. We call such a box-shaped neighborhood a good box.

Assume that we also have some *a priori* bounds

$$P_0 = [a_1, b_1] \times \ldots \times [a_m, b_m]$$

for our variables.

1.2 The algorithm

We want to find out how much can we shift the coordinates of p in order to get a good box. I.e. we want to find the box in the form

$$P_1 = [p_i - s_i^-, p_i + s_i^+] \times \ldots \times [p_m - s_i^-, p_m + s_i^+]$$

Denote $Q_0 = g(p)$.

We calculate the shifts using derivatives of F. Lets assume for a moment that $g(x)$ is linear in x. Than its derivative governs all the behavior. For example, if all coordinates of the derivative at p are positive and we decrease one of them, then the defectiveness also decrease. Since our g is nonlinear in general, this

works only in a small neighborhood of p. It is difficult to estimate the actual size of this neighborhood and we have coefficients **shiftCoef**, **shiftCoefLess** (they should be tuned by hand) to overcome this difficulty.

- $(F'(\alpha(p)))_i > 0$ in this case

$$s_i^+ = \frac{Q - Q_0}{\textbf{shiftCoef} \cdot (g')_i},$$

$$s_i^- = \frac{p_i - a_i}{\textbf{shiftCoefLess}};$$

- otherwise

$$s_i^+ = \frac{b_i - p_i}{\textbf{shiftCoefLess}};$$

$$s_i^- = \frac{Q - Q_0}{\textbf{shiftCoef} \cdot (g')_i}.$$

If g is linear then **all** the points from P_1 are good. In nonlinear situation we also can choose **shiftCoef** and **shiftCoefLess** big enough to ensure it. But this can lead to very small $s_i^{+,-}$ whereas we want them to be as big as possible.

1.3 The realization

The algorithm described above is realized using Matlab.

We are using finite difference derivatives in our calculations. Fix an index i of a variable. We choose a small number h_i such that $(p)_i + h_i$ and $(p)_i - h_i$ belong to $[a_i, b_i]$. Up to some technical details, we take

$$h_i = \frac{b_i - a_i}{\textbf{shiftSizeCoef}},$$

where **shiftSizeCoef** is a tunable constant.

Denote p^+ be p, with i'th coordinate changed to $p_i + h_i$, and p^- in a similar way. Consider a finite difference derivative

$$g'(p) = \frac{g(p^+) - g(p^-)}{2h_i}.$$

The bigger the **shiftSizeCoef**, the closer the finite difference derivative to a real one. But in highly nonlinear situation it may be helpful to choose it to be not too big in order to increase a volume of P_1. In this case the finite difference derivative should be understood as a Lipschitz constant along the corresponding coordinate of a mapping in a (not very small) neighborhood of p. Roughly speaking, the less the **shiftSizeCoef**, the bigger the norm of the finite difference derivative, the less volume will have P_1.

After we calculated P_1 we need to check, whether it is good or not. We use the standard Monte Carlo sampling to do it.

1.4 In our case

Let $i_{\mathbf{RNA}}$ be the index of the coordinate representing the concentration of RNA molecules and $i_{\mathbf{polyp}}$ be the index of the coordinate representing the concentration of polyprotein molecults. For $v \in \mathbb{R}^n$ denote

$$T(v) = 5v_{i_{\mathbf{RNA}}} + v_{i_{\mathbf{polyp}}}$$

For simplicity we explain only the case when we have only one type of inhibitor.

Assume that we have some data obtained either from real or from numerical observations. That is for the inhibitor be have a set of possible concentrations $C_i > 0,\ i = 1 \dots N_{\mathbf{inhC}}$. And there are inhibiton percentage $P_i \geq 0$ and a $D_i \geq 0$ deviation corresponding to each inhibitor concentration.

(in fact as an input data we use the power of concentration, so the concentration is equal to $3000 \cdot \mathbf{ki\_coef} \cdot 10^{power}$)

Let $\lambda(c)$ be variables values for the inhibitor concentration c.

$$g(\lambda(c)) = g_1(\lambda(c)) + g_2(\lambda(c)).$$

Here

$$g_1(c) = \sum_{i=1}^{N_{\mathbf{inhC}}} \frac{1}{D_i} \left(\frac{100T(\alpha(\lambda(c))(\mathbf{intTimeIng}))}{T(\alpha(\lambda(0)))} - P_i \right)^2$$

$$g_2(c) = \mathbf{wVesPen} \cdot e^{\mathbf{VEPS}|\mathbf{V0} - \mathbf{VOPT}|} - 1$$

Here **intTimeIng** is the integration time. g_2 adds penalty for a wrong number of vesicules – **V0** is the number of vesicles for zero concentration case, **VOPT** is the "true" number of vesicles and **wVesPen**, **VEPS** are tuning constants controlling the effect of the penalty.
