# Supplementary material for: A New Stochastic Model for Subgenomic Hepatitis C Virus Replication Considers Drug Resistant Mutants
Source: PLoS One. 2014 Mar 18;9(3):e91502. doi: 10.1371/journal.pone.0091502 (PMC3958367; doi:10.1371/journal.pone.0091502)
Supplement: Text S4 — Parameters optimization. (PDF) [file pone.0091502.s008.pdf]

1 Parameter estimation

1.1 Introduction

DEEP - Differential Evolution Entirely Parallel method is applied to the biological data fitting problem. The algorithm is an enhancement for the method developed recently [1, 2]. In order to increase the robustness of the procedure we implemented a new selection rule for Differential Evolution that considers several different objective functions in order to accept or reject an offspring to new generation. To reduce the computational complexity the population is not to be divided into branches, but instead, several oldest individuals are substituted with the same number of the best ones after predefined number of iterations. The individuals are selected on the basis of the number of generations, in which they survived without any change.

1.2 Formal problem statement

Let the vector $v(t,q) = (v_0(t,q), ..., v_{K-1}(t,q))^T$ describe the state of the system at time t. The dimension of v equals the number of state variables K. Let the vector $q = (q_0, ..., q_{I-1})^T$ be the vector of parameters with dimension I.

The system of ordinary differential equations of the first order in respect to the independent variable t and initial conditions describe the dynamics of the system:

$$\frac{dv}{dt} = f(v,q); \qquad v(0,q) = v_0; \tag{1.1}$$

The model parameters are to be estimated by fitting the model output to the experimental data. This is performed by minimizing the combined objective function that equals the sum of criteria that measure the deviation of the model from the desired behavior. We used the sum of squared differences between the data and model output as the main criterion:

$$\begin{aligned} F(v,q) &= \sum_{i=1}^{J}(v(t_i,q) - y(t_i))^T(v(t_i,q) - y(t_i)) + \sum_{l=1}^{L}\zeta_l P_l(q) \\ &= \varphi(v_1, ..., v_J) + \sum_{l=1}^{L}\zeta_l P_l(q) \to \min \\ v_i &= v(t_i,q), \end{aligned} \tag{1.2}$$

where J independent experimental observations are denoted $y(t_i) = (y_0(t), ..., y_{K-1}(t))^T$ and $i = 1, ..., J$ and P_l, $l = 1, ..., L$, are the additional criteria to be satisfied with weight coefficients ζ_l.

Constraints in the form of inequalities can be imposed for the subset of parameters:

$$q_i^{low} \leq q_i \leq q_i^{up} \quad i \in I_l. \tag{1.3}$$

1.3 Trigonometric transformation of constraints

Lets introduce new parameters u_i:

$$q_i = \alpha_i + \beta_i \ \sin\ (\eta u_i), \tag{1.4}$$

where scaling coefficient η is chosen experimentally, and

$$\alpha_i = (q_i^{up} + q_i^{low})/2; \quad \beta_i = (q_i^{up} - q_i^{low})/2.$$

Another transformation can be also used:

$$q_i = \alpha_i + \beta_i \ \tanh\ (\eta u_i), \tag{1.5}$$

Consequently, parameters q_i, $i \in I_l$ in (1.1) are substituted with there transformations (1.4) or (1.5) and minimization procedure is applied to determine unconstrained parameters u_i.

1.4 Differential Evolution Entirely Parallel method

Differential Evolution (DE) is a stochastic iterative optimization technique proposed by Storn and Price [5]. DE is an effective method for the minimization of various and complex objective functions. Its power is based on the fact that under appropriate conditions it will attain the global extremum of the functional; its weakness is in high computational demand and dependence on control variables, that provides a motivation to parallelize DE. Previous work in this area has produced a number of methods that perform well in certain particular problems, but not in general applications.

DE starts from the set of the randomly generated parameter vectors q_i, $i = 1, ..., NP$. The set is called population, and the vectors are called individuals. The population on each iteration is referred to as a generation. The size of population NP is fixed. The name of the method comes from the fact that the difference between the members of the current population are used in to generate offsprings (see Fig. 1).

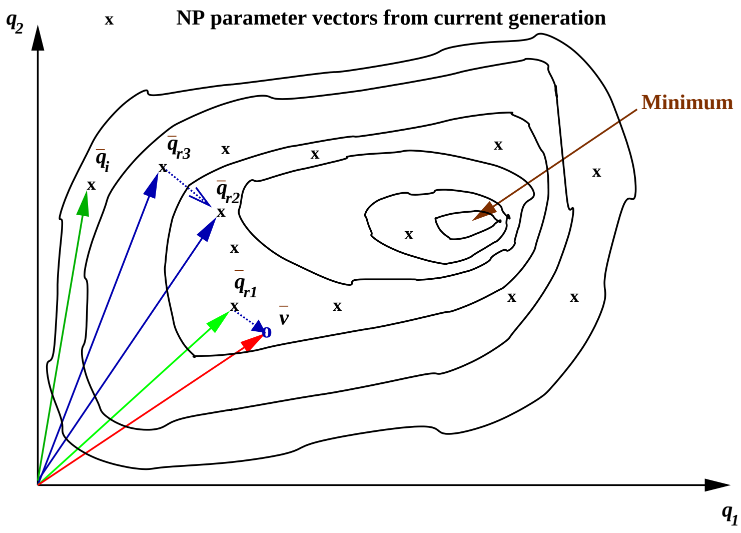

Figure 1: Geometric interpretation of Differential Evolution

1.5 Calculation of trial vectors

The first trial vector is calculated by:

$$v = q_{r1} + S(q_{r2} - q_{r3}) \tag{1.6}$$

where $q_\bullet$ is the member of the current generation g, S is a predefined scaling constant and $r1$, $r2$, $r3$ are different random indices of the members of population. The second trial vector is calculated using "trigonometric mutation rule" [6].

$$\begin{aligned} z &= \frac{q_{r1} + q_{r2} + q_{r3}}{3} + (\varphi_2 - \varphi_1)(q_{r1} - q_{r2}) \\ &+ (\varphi_3 - \varphi_2)(q_{r2} - q_{r3}) + (\varphi_1 - \varphi_3)(q_{r3} - q_{r1}) \end{aligned} \tag{1.7}$$

where $\varphi_i = |F(q_{ri})|/\varphi^*$, $i = 1, 2, 3$, $\varphi^* = |F(q_{r1})| + |F(q_{r2})| + |F(q_{r3})|$. The third trial vector is defined as follows:

$$w_j = \begin{cases} v_j, & j = \langle n \rangle_I, \langle n+1 \rangle_I, ..., \langle n+L-1 \rangle_I \\ z_j & j < \langle n \rangle_I \; OR \; j > \langle n+L-1 \rangle_I \end{cases} \tag{1.8}$$

where n is a randomly chosen index, $\langle x \rangle_y$ is the reminder of division x by y and L is determined by $Pr(L = a) = (p)^a$ where p is the probability of crossover.

The process is illustrated in Fig. 2.

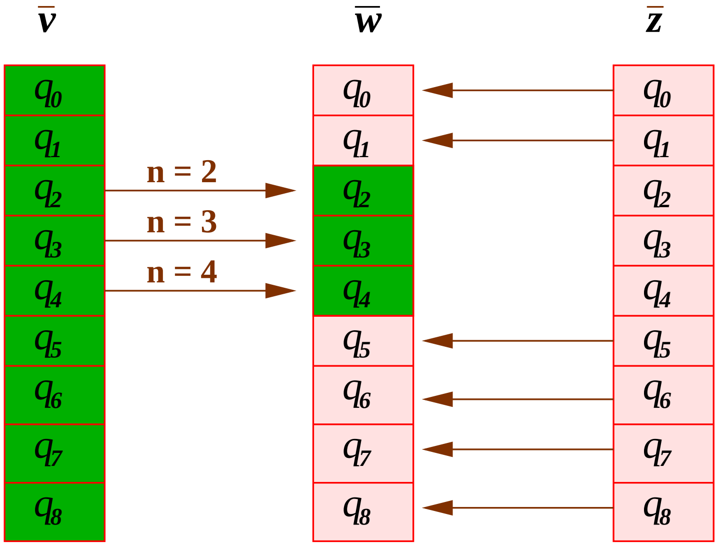

Figure 2: Construction of the trial vector

1.6 Preserving population diversity

An efficient adaptive scheme for selection of internal parameters S and p based on the control of the population diversity was proposed in [7]:

$$var_j = \frac{1}{NP} \sum_{i=0}^{NP-1} \left(q_{i,j} - \frac{1}{NP} \sum_{k=0}^{NP-1} q_{k,j} \right)^2 \tag{1.9}$$

where $j = 0, ..., I-1$. Then

$$S_j = \begin{cases} \sqrt{\frac{NP \cdot (c_j - 1) + p_j(2-p_j)}{2 \cdot NP \cdot p_j}} & NP \cdot (c_j - 1) + p_j(2-p_j) \geq 0 \\ S_{inf} & NP \cdot (c_j - 1) + p_j(2-p_j) < 0 \end{cases} \tag{1.10}$$

and

$$p_j = \begin{cases} -(NP \cdot S_j^2 - 1) + \sqrt{(NP \cdot S_j^2 - 1)^2 - NP \cdot (1 - c_j)} & c_j \geq 1 \\ p_{inf} & c_j < 1 \end{cases} \tag{1.11}$$

and a new control parameter γ was introduced:

$$c_j^{new} = \gamma \left(var_j / var_j^{new} \right) \tag{1.12}$$

1.7 Parallelization

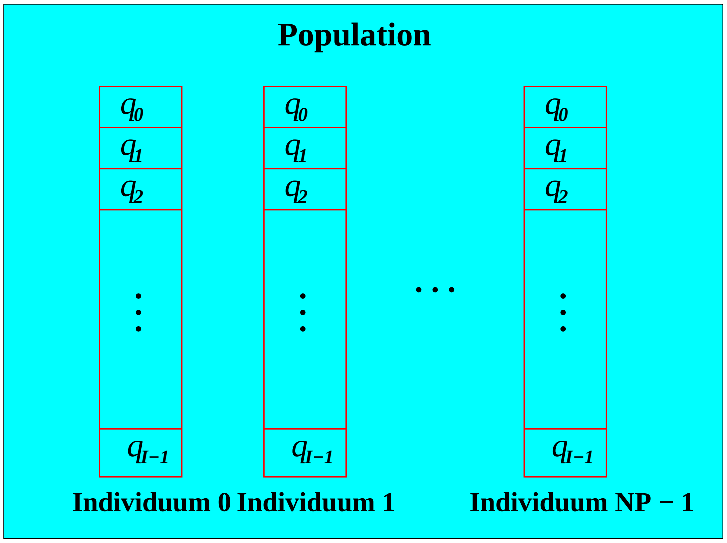

Figure 3: Differential Evolution processes the population of individuals

Being an evolutionary algorithm, DE can be easily parallelized due to the fact that each member of the population is evaluated individually. In the method reported earlier [1, 2, 3] the whole population is to be divided into branches. The information exchange between branches allowed the best member of the branch to substitute the oldest member of another branch after each Π iterations. It turned out that in biological applications the value of Π was considerably small, making branches an unnecessary complication.

In the latest version the branches are eliminated, and several oldest members of the population are substituted by the same number of individuals, having the best values of objective function. The age of an individual in our approach is defined by the number of iterations, in which the individual survived without changes. The number of seeding individuals Ψ and the number of iterations called seeding interval Θ are the new parameters of the algorithm. The fact that the certain parameter set has not been update during several iterations indicates that this set corresponds to the local minimum of the objective function. As we seek the global minimum such parameter set can be deleted from the population. The set of parameters that corresponds to the minimal functional value found so far in parallel branch is copied in

place of the deleted parameter set in target branch.

One of the parameters of the algorithm determines the number of parallel threads used to calculate the objective function. We utilized the Thread Pool API from GLIB project [8] and construct the pool with the defined number of worker threads. The calculation of objective function for each trial vector is pushed to the asynchronous queue. The calculation starts as soon as there is an available thread. The thread synchronization condition is determined by the fact that objective function is to be calculated once for each individual in the population on each iteration.

1.8 New Selection Rule

Algorithm 1 SELECTION

```
proc select (individual) =
{
if (F < the value of the parent) then
  Accept offspring
else
  for all additional criteria P do
    if (P < the value of the parent) then
      Generate the random number U.
      if (U < control parameter for this criterion) then
        Accept offspring
      end if
    end if
  end for
end if
}
```

In order to increase the robustness of the procedure we have implemented a new selection rule for DE (Algorithm 1). An offspring is to be accepted to the new generation in accordance with several different criteria. The offspring replaces its parent if the value of the combined objective function for the offspring set of parameters is less than that for the parental one. The additional criteria are checked in the opposite case. The offspring replaces its parent if the value of some criterion is better, and a randomly selected value is less than the predefined parameter for this criterion.

1.9 Stopping criterion

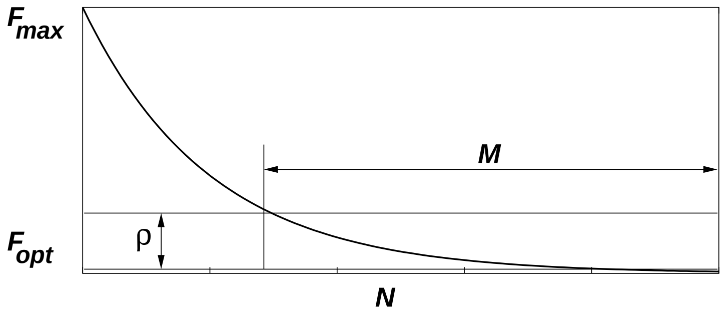

Figure 4: Stopping criterion. The value of objective function F is plotted against the number of iterations N

Calculations are stopped in case that the functional F decreases less than a predefined value ρ during M steps, Fig. 4.

1.10 Implementation

New algorithm was implemented in C programming language as the software framework with suitable interface that allows a user to express the objective function using different computer systems widely used in biomedical applications, such as Octave, R or KNIME. The control parameters of the algorithm are defined in the datafile that uses an INI-format. The framework is operated via the simple command line interface. The Enhanced DEEP method can be embedded in new software. The developed new software is available on the project site [4].

Runs were performed with different combinations of parameters on the cluster (1980 Intel Xeons) in the Joint Supercomputer Center of the Russian Academy of Sciences, Moscow.

2 Parameter Values

Table 1: **Parameter sets: 10 sets of parameter values that optimize the combined objective function. Mean value and standard deviation are denoted μ and σ respectively. Endpoints of the intervals for confidence levels 50%, 95% and 99% are given in the last three rows. Lower and upper limits obtained by bootstraping using 1000 replicas are denoted lo and hi respectively.**

#	kp	kv	mp	mpolyp	kout_v	kcf	mcf
1	2.999986	3.000000	0.823785	0.429819	1.114557	5.000005	1.0005
2	2.999678	2.729485	0.820095	0.427777	1.113277	5.001416	1.0050
3	2.999404	3.000000	0.865871	0.437008	1.129952	5.000008	1.0003
4	2.949448	1.911609	1.000000	0.452166	1.177727	5.000793	1.0005
5	2.998484	2.777688	0.833380	0.431802	1.118594	5.000709	1.0000
6	2.999953	2.999547	1.000000	0.434513	1.155443	5.000000	1.0000
7	2.997663	2.999879	0.850291	0.429821	1.120177	5.000003	1.0000
8	2.926132	3.000000	0.549895	0.425220	1.061066	5.066627	1.0064
9	3.000000	3.000000	0.944671	0.439802	1.148911	5.000001	1.0000
10	2.999995	2.994594	0.851762	0.430848	1.121471	5.000008	1.0012
μ	2.987074	2.841280	0.853975	0.433878	1.126118	5.006957	1.0014
σ	0.026561	0.342427	0.127358	0.007734	0.031094	0.020972	0.0023
50%	0.005665	0.073037	0.027164	0.001650	0.006632	0.004473	0.0005
95%	0.016462	0.212234	0.078936	0.004794	0.019272	0.012998	0.0014
99%	0.021635	0.278923	0.103739	0.006300	0.025327	0.017082	0.0019
lo	2.955551	2.433021	0.704967	0.428236	1.098155	5.000002	1.0000
hi	2.999963	2.999976	0.964388	0.441586	1.156975	5.027226	1.0041

The ensemble of parameter sets is presented in Table 1 and 2.

The scatter plot 5 shows the low variability for the majority of the parameters.

Table 2: **Parameter sets: 6 sets of parameters related to mutants. Mean value and standard deviation are denoted μ and σ respectively. Endpoints of the intervals for confidence levels 50%, 95% and 99% are given in the last three rows. Lower and upper limits obtained by bootstraping using 1000 replicas are denoted lo and hi respectively.**

#	kcm_{1b}	kcm_{2b}	kcm_{1v}	kcm_{2v}	$kcm_{3b,v}$	kim_{1b}	kim_{2b}	kim_{3b}	kim_{1v}	kim_{2v}	kim_{3v}
1	0.800	0.700	0.800	0.700	0.400	30.0	200.0	600.0	15.0	13.0	80.0
2	0.750	0.642	0.861	0.746	0.539	29.3	155.8	782.7	16.4	10.2	121.3
3	0.870	0.726	0.724	0.657	0.405	30.0	197.0	580.1	13.4	10.0	78.8
4	0.731	0.611	0.799	0.615	0.314	27.4	179.8	772.3	13.6	10.5	86.4
5	0.736	0.605	0.814	0.600	0.469	38.3	191.9	470.9	13.8	17.1	121.6
6	0.805	0.800	0.718	0.664	0.312	23.6	212.4	579.9	11.2	19.9	71.4
μ	0.782	0.681	0.786	0.664	0.407	29.8	189.5	631.0	13.9	13.4	93.2
σ	0.053	0.076	0.055	0.054	0.088	4.851	19.63	122.3	1.72	4.17	22.4
50%	0.015	0.021	0.015	0.015	0.024	1.336	5.406	33.67	0.47	1.15	6.2
95%	0.043	0.061	0.044	0.043	0.071	3.882	15.71	97.85	1.38	3.34	17.8
99%	0.056	0.080	0.058	0.057	0.093	5.101	20.65	128.6	1.81	4.39	23.5
lo	0.733	0.607	0.733	0.608	0.313	25.3	167.2	507.3	11.9	10.1	73.9
hi	0.837	0.783	0.835	0.738	0.504	35.6	206.5	774.1	15.9	19.0	121.5

References

[1] Kozlov Konstantin, Samsonov Alexander. DEEP – Differential Evolution Entirely Parallel Method for Gene Regulatory Networks, PaCT '09: Proceedings of the 10th International Conference on Parallel Computing Technologies. Berlin, Heidelberg: Springer-Verlag, 126–132 (2009)

[2] Kozlov, K. and Samsonov, A., DEEP – Differential Evolution Entirely Parallel Method for Gene Regulatory Networks. *Journal of Supercomputing*, 57:172–178 (2011)

[3] Konstantin Kozlov, Svetlana Surkova, Ekaterina Myasnikova, John Reinitz, Maria Samsonova. Modeling of gap gene expression in Drosophila Kruppel mutants, PLoS Computational Biology, 8(8):e1002635 (2012)

[4] Differential Evolution Entirely Parallel method http://urchin.spbcas.ru/trac/DEEP

[5] Storn R., Price K., Differential Evolution – A Simple and Efficient Heuristic for Global Optimization over Continuous Spaces, Technical Report TR-95-012, ICSI (1995)

[6] Hui-Yuan Fan and Jouni Lampinen, A Trigonometric Mutation Operation to Differential Evolution, Journal of Global Optimization 27: 105–129 (2003)

[7] Zaharie D., Parameter Adaptation in Differential Evolution by Controlling the Population Diversity, in D.Petcu et al. (eds), Proc. of 4th InternationalWorkshop on Symbolic and Numeric Algorithms for Scientific Computing, Timisoara, Romania, 385–397 (2002)

[8] GLib http://developer.gnome.org/glib/

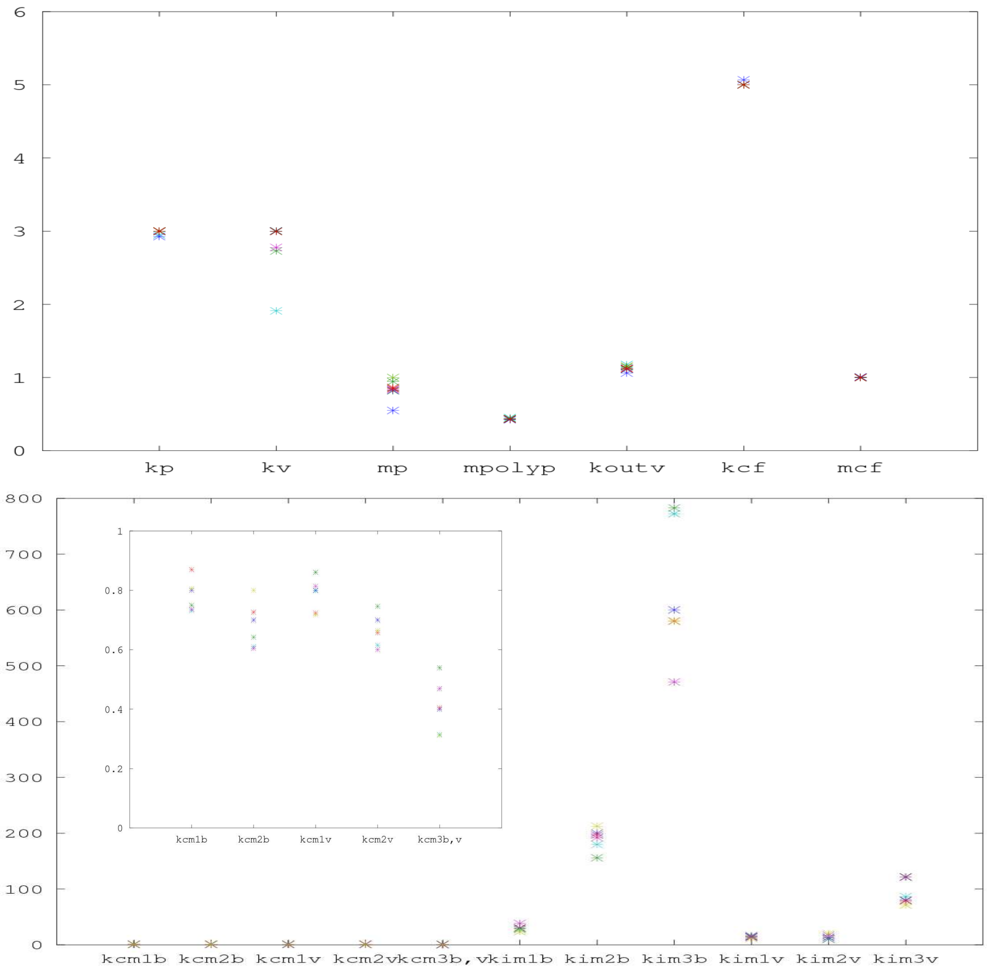

Figure 5: Scatter plot for all parameters
